# Functional Redundancy of Two Pax-Like Proteins in Transcriptional Activation of Cyst Wall Protein Genes in *Giardia lamblia*

**DOI:** 10.1371/journal.pone.0030614

**Published:** 2012-02-15

**Authors:** Shen-Fung Chuang, Li-Hsin Su, Chao-Cheng Cho, Yu-Jiao Pan, Chin-Hung Sun

**Affiliations:** Department of Parasitology, College of Medicine, National Taiwan University, Taipei, Taiwan, Republic of China; University of Oklahoma Health Sciences Center, United States of America

## Abstract

The protozoan *Giardia lamblia* differentiates from a pathogenic trophozoite into an infectious cyst to survive outside of the host. During encystation, genes encoding cyst wall proteins (CWPs) are coordinately induced. Pax family transcription factors are involved in a variety of developmental processes in animals. Nine Pax proteins have been found to play an important role in tissue and organ development in humans. To understand the progression from primitive to more complex eukaryotic cells, we tried to identify putative *pax* genes in the *G. lamblia* genome and found two genes, *pax1* and *pax2*, with limited similarity. We found that Pax1 may transactivate the encystation-induced *cwp* genes and interact with AT-rich initiatior elements that are essential for promoter activity and transcription start site selection. In this study, we further characterized Pax2 and found that, like Pax1, Pax2 was present in *Giardia* nuclei and it may specifically bind to the AT-rich initiator elements of the encystation-induced *cwp1-3* and *myb2* genes. Interestingly, overexpression of Pax2 increased the *cwp1-3* and *myb2* gene expression and cyst formation. Deletion of the C-terminal paired domain or mutation of the basic amino acids of the paired domain resulted in a decrease of nuclear localization, DNA-binding activity, and transactivation activity of Pax2. These results are similar to those found in the previous Pax1 study. In addition, the profiles of gene expression in the Pax2 and Pax1 overexpressing cells significantly overlap in the same direction and ERK1 associated complexes may phosphorylate Pax2 and Pax1, suggesting that Pax2 and Pax1 may be downstream components of a MAPK/ERK1 signaling pathway. Our results reveal functional redundancy between Pax2 and Pax1 in up-regulation of the key encystation-induced genes. These results illustrate functional redundancy of a gene family can occur in order to increase maintenance of important gene function in the protozoan organism *G. lamblia*.

## Introduction

*Giardia lamblia* is a common intestinal protozoan parasite responsible for outbreaks of waterborne diarrhea [1], [2], [3]. Giardiasis is prevalent in developing countries of the tropics due to poor hygiene [4], [5]. It is also associated with epidemic outbreaks of diarrheal disease due to water resource contamination in developed countries [4], [5]. Most infections are asymptomatic, but patients with giardiasis may have gastrointestinal symptoms or may have a post-giardiasis irritable bowel syndrome [6], [7]. Chronic giardiasis in children may lead to malabsorption, weight loss, and delayed mental development [8].

*G. lamblia* has two life cycle stages- a trophozoite form that parasitizes the human small intestine and a cyst form that persists in the hostile environment [9], [10], [11]. *Giardia* trophozoites colonizing the upper intestinal tract must successfully encyst in order to infect a new host. During encystation, an extracellular cyst wall is synthesized, protecting the parasite from hypotonic lysis by fresh water and gastric acid and thereby helping transmission [1], [2]. While the entire life cycle can be reproduced *in vitro*, the pathogenesis and molecular mechanisms involved in regulation of cyst wall synthesis are poorly understood. Expression of three cyst wall structural proteins (CWP1, CWP2, and CWP3) and enzymes in the cyst wall polysaccharide biosynthetic pathway is coordinately induced during encystation [12], [13], [14], [15], [16], [17]. *G. lamblia* may respond to encystation stimuli via activation of signal transduction pathways that are involved in the regulation of synthesis of CWPs and polysaccharides. Extracellular signal-related kinase 1 and 14-3-3 protein may be involved in encystation-induced signal transduction pathways [18], [19], [20].

*G. lamblia* is classified as a single-celled protozoan eukaryote. It has many special features that are biologically different from those of higher eukaryotes [2], [21]. Very simplified machineries for many cellular processes, including DNA synthesis, transcription and RNA processing, have been identified in its genome, suggesting that *Giardia* may have diverged early and that the missing components may be nonessential or too divergent [21]. Only four of the twelve general transcription initiation factors have giardial homologs [21], [22]. Many giardial transcription factors, including TATA binding protein, appear to have diverged at a higher rate than those of crown group eukaryotes [22]. *Giardia* does not have some components of multisubunit mediators that bridges transcriptional activators or repressors to basal RNA polymerase II initiation machinery [23]. Giardial RNA polymerase II has no regular heptad repeats in the carboxyl-terminal domain and transcription by RNA polymerase II is highly resistant to α-amanitin [22], [24]. The giardial promoter regulatory mechanism may be unusual because unusually short 5′-flanking regions (<65 bp) are sufficient for the expression of many giardial protein-coding genes [12], [13], [15], [25], [26], [27]. Within the short promoter regions, no consensus TATA boxes or other *cis*-acting elements characteristic of higher eukaryotic promoters have been observed [12], [13], [15], [25], [26], [27], [28]. Instead, AT-rich sequences that are functionally similar to the initiator (Inr) element in higher eukaryotes have been found around the transcription start sites of many genes [12], [13], [14], [15], [25], [26], [27], [28], [29], [30], [31].

Because of the importance of the cyst stage, many researches focus on identifying the key transcription factors regulating cyst wall synthesis. Several transcription factors that have been characterized to date are up-regulated and involved in *cwp* gene regulation during encystation [18], [29], [31], [32], [33], [34]. A Myb family transcription factor (Myb2) may bind to the promoters of four key encystation-induced genes, *cwp1-3*, and *myb2* itself, suggesting that Myb2 may be involved in co-ordinating their differential expression [29], [33]. A GARP family transcription factor may be involved in transcriptional regulation of many different genes including the encystation-induced *cwp1* gene and constitutive *ran* gene [32]. A WRKY family transcription factor can bind to specific sequences in the *cwp1*, *cwp2*, and *myb2* promoters and upregulate expression of these genes [18]. An E2F family transcription factor can transactivate the *thymidine kinase* and *cwp1* promoters to increase the expression of these proteins and cyst formation [35]. An AT-rich interaction domain (ARID)-family transcription factor can bind to specific AT-rich Inr sequences of *cwp1-3* gene promoters and up-regulate the *cwp1* gene [31]. In addition, a Pax protein (Pax1) has been identified in *Giardia* and it can also bind to specific AT-rich Inr sequences of *cwp1-3* and *myb2* gene promoters and function as an important transactivator in the regulation of the *cwp1-3* and *myb2* genes [31]. It is interesting that *G. lamblia* possesses the transcription factors identified in both plants and animals (Myb, ARID, and E2F) or the transcription factors identified only in plants (WRKY and GARP) and animals (Pax) [34].

Pax (paired box) proteins have been found in *Drosophila*, *Caenorhabditis elegans*, sea urchin, zebrafish, chicken, mouse, and human, but not in plants [36], [37], [38], [39], [40], [41], [42]. Pax proteins play important roles in the development of many tissues and organs in mammals, including muscle, thymus, thyroid, pancreas, neuron, eyes, and kidney [37], [38], [39]. Some Pax proteins also play important roles in differentiation of the neural-crest cells, myoblast cells and B cells [37], [38], [39]. In addition, mutation of the *pax* genes may lead to developmental defects and abnormal overexpression of the *pax* genes may lead to tumor formation [41]. Pax proteins contain a 128-amino acid DNA-binding domain, the paired domain [37], [38], [39], [43]. In addition to the paired domain, some Pax proteins contain a homeodomain, which is also capable of binding DNA [42], [44]. Nine *pax* genes have been found in mammals [37], [38], [39], [42]. These Pax proteins can be classified on the basis of the presence of a partial homeodomain or full homeodomain and an octapeptide. Most Pax proteins have an octapeptide, except Pax4/6 proteins [42], [44]. Pax proteins function in sequence-specific DNA binding and they may mediate transcriptional activation or repression. Pax binding sequences can have a (G/T)T(T/C)(C/A)(C/T)(G/C)(G/C) sequence and the Pax protein with a homeodomain may bind to an ATTA sequence [37], [38], [39], [42], [45]. Pax may also interact with other transcription factors to regulate the target promoters [39] and function as transcriptional activators or repressors [46], [47], [48].

We found two *pax*-like genes (*pax1* and *pax2*) encoding proteins with paired domains in *G. lamblia* genome database [34]. We have determined the function of Pax1 in *Giardia* [34]. Since many of the Pax proteins have been implicated in important biological processes in higher eukaryotes [37], [38], [39], [40], [41], [42], *G. lamblia* might use Pax proteins to adapt it to specific differentiation processes, such as encystation. In this study, we found that Pax2 and Pax1 have limited sequence similarity. However, the function of Pax2 is similar to Pax1 and it can bind to specific AT-rich Inr sequences and function as a transactivator of the *cwp1-3* and *myb2* genes to regulate *G. lamblia* differentiation into dormant cysts. We also found that Pax2 and Pax1 can regulate similar profiles of gene expression. Little is known about encystation-induced signal transduction pathways that are involved in the regulation of CWP synthesis. A member of the MAPK family, extracellular signal-related kinase 1 (ERK1), has been identified to exhibit a significantly increased kinase activity during encystation [18], [49]. We found that Pax2 and Pax1 can be phosphorylated by ERK1 associated complexes, suggesting that Pax2 and Pax1 may be down-stream components of a MAPK/ERK1 signaling pathway. Our results suggest that both Pax2 and Pax1 may be an important transcription factor regulating differentiation in the protozoan pathogen, *G. lamblia*. The studies of *G.iardia* Pax proteins may help to understand the progression of the control of gene expression from primitive to more complex eukaryotic cells.

## Methods

### *G. lamblia* culture

Trophozoites of *G. lamblia* WB (ATCC 50803), clone C6, were cultured in modified TYI-S33 medium [50]. Encystation was performed as previously described [14]. Briefly, trophozoites that were grown to late log phase in growth medium were harvested and encysted for 24 h in TYI-S-33 medium containing 12.5 mg/ml bovine bile at pH 7.8 at a beginning density of 5×10^5^ cells/ml.

### Cyst count

The cyst count was performed on the stationary phase cultures (∼2×10^6^ cells/ml) during vegetative growth as previously described [51]. Cells were subcultured in growth medium with suitable selection drugs at an initial density of 1×10^6^ cells/ml. Cells seeded at this density became confluent within 24 h. Confluent cultures were maintained for an additional 8 h to ensure that the cultures were in stationary phase (at a density of∼2×10^6^ cells/ml). The cyst count was performed on these stationary phase cultures. Cultures were chilled and cells were washed twice in double-distilled water at 4°C and trophozoites were lysed by incubation in double-distilled water overnight at 4°C. Cysts were washed three times in double-distilled water at 4°C. Water-resistant cysts were counted in a hemacytometer chamber. The cyst count was also performed on 24 h encysting cultures.

### Isolation and analysis of the *pax2* gene

The *G. lamblia* genome database (http://www.giardiadb.org/giardiadb/) [21], [52] was searched with the amino acid sequences of the paired domain of *Drosophila* Pox meso (GenBank accession number **NM_001043222**) using the BLAST program [53]. This search detected two putative Pax homologues (open reading frames 32686 (Pax1) and 16640 (Pax2) in the *G. lamblia* genome database). The GenBank accession numbers for the giardial Pax1 and Pax2 are XM_001704983.1 and XM_001709076.1, respectively. The Pax2 coding region with 300 nt of 5′- flanking regions was cloned and the nucleotide sequence was determined. The *pax2* gene sequence in the database was correct. To isolate the cDNA of the *pax2* gene, we performed RT-PCR with *pax2*-specific primers using total RNA from *G. lamblia*. For RT-PCR, 5 µg of DNase-treated total RNA from vegetative and 24 h encysting cells was mixed with oligo (dT)12-18 and random hexamers and Superscript II RNase H- reverse transcriptase (Invitrogen). Synthesized cDNA was used as a template in subsequent PCR with primers Pax2F and Pax2R. Oligonucleotides used in this study are listed in Table S1. Genomic and RT-PCR products were cloned into pGEM-T easy vector (Promega) and sequenced (Applied Biosystems, ABI).

### RNA extraction, RT-PCR and quantitative real-time PCR analysis

Total RNA was extracted from *G. lamblia* cell line at the differentiation stages indicated in figure legends using TRIzol reagent (Invitrogen). cDNA was synthesized as described above. Semi-quantitative RT-PCR analysis of *pax2* (**XP_001709128**, open reading frame 16640), *pax2-ha*, *cwp1* (**U09330**, open reading frame 5638), *cwp2* (**U28965**, open reading frame 5432), *cwp3* (**AY061927**, open reading frame 2421), *myb2* (**AY082882**, open reading frame 8722), *ran* (**U02589**, open reading frame 15869), and *18S ribosomal RNA* (**M54878**, open reading frame r0019) gene expression was performed using primers Pax2F and Pax2R, Pax2F and Pax2HAR, cwp1F and cwp1R, cwp2F and cwp2R, cwp3F and cwp3R, myb2F and myb2R, ranF and ranR, 18SrealF and 18SrealR, respectively. For quantitative real-time PCR, SYBR Green PCR master mixture was used (Kapa Biosystems). PCR was performed using an Applied Biosystems PRISMTM 7900 Sequence Detection System (Applied Biosystems). Specific primers were designed for detection of the *pax2*, *pax2-ha*, *cwp1*, *cwp2*, *cwp3*, *myb2*, *ran*, and *18S ribosomal RNA* genes: Pax2realF and Pax2realR; Pax2HAF and Pax2HAR; cwp1realF and cwp1realR; cwp2realF and cwp2realR; cwp3realF and cwp3realR; myb2realF and myb2realR; ranrealF and ranrealR;18SrealF and 18SrealR. The results are expressed as relative expression level over control. Student's *t*-tests were used to determine statistical significance of differences between samples.

### Plasmid construction

All constructs were verified by DNA sequencing with a BigDye Terminator 3.1 DNA Sequencing kit and an Applied Biosystems 3100 DNA Analyser (Applied Biosystems). Plasmid 5′Δ5N-Pac was a gift from Dr. Steven Singer and Dr. Theodore Nash [54]. The *pax2* gene and its 300-bp 5′-flanking region was amplified with oligonucleotides Pax2XF and Pax2MR, digested with XbaI/MluI, and cloned into NheI/MluI-digested pPop2NHA [55]. The resulting plasmid, pPPax2, contained the *pax2* gene controlled by its own promoter with an HA tag fused at its C-terminus. For constructing pPPax2m1, a PCR with oligonucleotides Pax2XF and Pax2m1R generated a 0.9-kb product. Another PCR with primers Pax2m1F and Pax2MR generated a 0.4-kb PCR product. A second run of PCR with the above two products and primers Pax2XF and Pax2MR generated a 1.3-kb PCR product that was digested with XbaI/MluI, and cloned into NheI/XbaI-digested pPop2NHA [55]. The resulting plasmid, pPPax2m1, contains a *pax2* gene with a mutation of the coding region of a stretch of basic amino acids between residues 185 and 205 which is located inside the paired domain. For constructing pPPax2m2, a PCR with oligonucleotides Pax2XF and Pax2m2R generated a 1.1-kb product. Another PCR with primers Pax2m2F and Pax2MR generated a 0.2-kb PCR product. A second run of PCR with the above two products and primers Pax2XF and Pax2MR generated a 1.3-kb PCR product that was digested with XbaI/MluI, and cloned into NheI/MluI-digested pPop2NHA [55]. The resulting plasmid, pPPax2m2, contains a *pax2* gene with a mutation of the coding region of a stretch of basic amino acids between residues 226 and 248, which is located inside the paired domain. For constructing pPPax2m3, a PCR product generated with oligonucleotides Pax2XF and Pax2m3MR was digested with XbaI and MluI and cloned into NheI/MluI-digested pPop2NHA [55]. The resulting plasmid, pPPax2m3, contains a *pax2* gene lacking the paired domain and the C-terminal 3 amino acids (residues 172–302).

### Transfection and Western blot analysis

Cells transfected with the pP series plasmid containing the *pac* gene were selected and maintained with 54 µg/ml puromycin. Western blots were probed with anti-V5-horseradish peroxidase (HRP) (Invitrogen), anti-HA monoclonal antibody (Covance, Princeton, NJ; 1/5000 in blocking buffer), anti-RAN antibody (1/10000 in blocking buffer), anti-α tubulin antibody (Sigma, 1/1500 in blocking buffer) [56], anti-CWP1 antibody (1/10000 in blocking buffer) [33], or anti-Pax2 (1/10000 in blocking buffer), and detected with HRP-conjugated goat anti-mouse IgG (Pierce, 1/5000 in blocking buffer) or HRP-conjugated goat anti-rabbit IgG (Pierce, 1/5000) and enhanced chemiluminescence (GE Healthcare).

### Expression and purification of recombinant Pax2 protein

The genomic *pax2* gene was amplified using oligonucleotides Pax2F and Pax2R. The product was cloned into the expression vector pET101/D-TOPO (Invitrogen) in frame with the C-terminal His and V5 tag to generate plasmid pPax2. To make the pPax2m1 (or pPax2m2) expression vector, the *pax2* gene was amplified using two primer pairs Pax2F and Pax2m1R (or Pax2m2R), and Pax2m1F (or Pax2m2F) and Pax2R. The two PCR products were purified and used as templates for a second PCR. The second PCR reaction also included primers Pax2F and Pax2R, and the product was cloned into the expression vector to generate plasmid pPax2m1 (or pPax2m2). To make the pPax2m3 expression vector, the *pax2* gene was amplified using two primers Pax2F and Pax2m3R. The product was cloned into the expression vector to generate plasmid pPax2m3. The pPax2, pPax2m1, pPax2m2, or pPax2m3 plasmid was freshly transformed into *Escherichia coli* BL21 Star™(DE3) (Invitrogen). An overnight pre-culture was used to start a 250-ml culture. *E. coli* cells were grown to an A600 of 0.5, and then induced with 1 mM isopropyl-D-thiogalactopyranoside (Promega) for 4 h. Bacteria were harvested by centrifugation and sonicated in 10 ml of buffer A (50 mM sodium phosphate, pH 8.0, 300 mM NaCl) containing 10 mM imidazole and protease inhibitor mixture (Sigma). The samples were centrifuged, and the supernatant was mixed with 1 ml of 50% slurry of nickel-nitrilotriacetic acid Superflow (Qiagen). The resin was washed with buffer A containing 20 mM imidazole and eluted with buffer A containing 250 mM imidazole. Fractions containing Pax2, Pax2m1, Pax2m2, or Pax2m3 were pooled, dialyzed in 25 mM HEPES pH 7.9, 20 mM KCl, and 15% glycerol, and stored at −70°C. Protein purity and concentration were estimated by Coomassie Blue and silver staining compared with serum albumin. Pax2, Pax2m1, Pax2m2, or Pax2m3 was purified to apparent homogeneity (>95%).

#### Generation of anti-Pax2 antibody

Purified Pax2 protein was used to generate rabbit polyclonal antibodies through a commercial vendor (Angene, Taipei, Taiwan).

### Generation of anti-RAN antibody

The genomic *ran* gene was amplified using oligonucleotides RanF and RanR and the product was cloned into the expression vector pET101/D-TOPO (Invitrogen) in frame with the C-terminal His and V5 tags to generate plasmid pRan. The pRan plasmid was freshly transformed into *E. coli* BL21 Star™(DE3) (Invitrogen). RAN was purified to apparent homogeneity (>95%) using the same purification method as described above. Purified RAN protein was used to generate rabbit polyclonal antibodies through a commercial vendor (Angene, Taipei, Taiwan).

### Immunofluorescence assay

The pPPax2, pPPax2m1, pPPax2m2, or pPPax2m3 stable transfectants were cultured in growth medium under puromycin selection. Cells cultured in growth medium or encystation medium for 24 h were harvested, washed in phosphate-buffered saline (PBS), and attached to glass coverslips (2×10^6^ cells/coverslip) and then fixed and stained [15]. Cells were reacted with anti-HA monoclonal antibody (1/300 in blocking buffer; Molecular Probes) and anti-mouse ALEXA 488 (1/500 in blocking buffer, Molecular Probes) as the detector. ProLong antifade kit with 4′,6-diamidino-2-phenylindole (Invitrogen) was used for mounting. Pax2, Pax2m1, Pax2m2, or Pax2m3 was visualized using a Leica TCS SP5 spectral confocal system.

### Electrophoretic Mobility Shift Assay

Double-stranded oligonucleotides specified throughout were 5′-end-labeled as described [26]. Binding reaction mixtures contained the components described [31]. Labeled probe (0.02 pmol) was incubated for 15 min at room temperature with 5 ng of purified Pax2, Pax2m1, Pax2m2, or Pax2m3 protein in a 20 µl volume supplemented with 0.5 µg of poly (dI-dC) (Sigma). Competition reactions contained 200-fold molar excess of cold oligonucleotides. In an antibody supershift assay, 0.8 µg of an anti-V5 antibody (Bethyl Laboratories) was added to the binding reaction mixture. The mixture was separated on a 6% acrylamide gel by electrophoresis.

### Co-immunoprecipitation assay

Plasmid pPERK1 or pPERK1m has been described previously [18]. The 5′Δ5N-Pac, pPERK1, and pPERK1m stable transfectants were inoculated into encystation medium (5×10^7^ cells in 45 ml medium) and harvested after 24 h in encystation medium under drug selection and washed in phosphate-buffered saline. Cells were lysed in luciferase lysis buffer (Promega) and protease inhibitor (Sigma) and then vortexed with glass beads. The cell lysates were collected by centrifugation and then incubated with anti-HA antibody conjugated to beads (Bethyl Laboratories Inc.). The beads were washed three times with luciferase lysis buffer (Promega). Finally the beads were then resuspended in sample buffer and analyzed by Western blot and probed with anti-HA monoclonal antibody (1/5000 in blocking buffer; Sigma), anti-Pax2 (1/10000 in blocking buffer), and anti-Pax1 (1/10000 in blocking buffer), and detected with HRP-conjugated goat anti-mouse IgG (Pierce, 1/5000) or HRP-conjugated goat anti-rabbit IgG (Pierce, 1/5000) and enhanced chemiluminescence (GE Healthcare).

### Kinase assay

Kinase assays were performed as described with modification [57]. IP-kinase assays were performed using the 5′▵5N-Pac, pPERK1, or pPERK1m stable transfectants cultured in growth or encystation medium for 24 h and anti-HA antibody for immunoprecipitation. *Giardia* trophozoites (∼10^8^cells) were harvested by centrifugation and lysed in buffer X (25 mM Tris-HCl, pH 7.5, 10 mM EDTA, 10 mM EGTA, 20 mM NaCl, 10% glycerol, 1 mM dithiothreitol, 20 mM β-glycerophosphate, 1 mM sodium *o*-vanadate, 1 mM NaF, 1% Triton X-100, and 1% Nonidet P-40) containing glass beads and complete protease inhibitor cocktail (Roche). The samples were centrifuged and the concentration of the supernatant was estimated by SDS-PAGE. The supernatant was mixed and rotated with anti-HA beads (Covance) at 4°C for 2 h. Beads were washed four times with buffer X and twice with kinase buffer (50 mM HEPES-KOH, pH 7.5, 20 mM MgCl2, 5 mM EGTA, 1 mM dithiothreitol, 20 mM β-glycerophosphate, and 1 mM sodium *o*-vanadate). The beads were mixed in a reaction mixture containing 50 µM ATP, and 10 µCi of γ-^32^P-ATP in kinase buffer. Recombinant Pax2 or Pax1 protein (4 µg) was added to the reaction mixture and incubated for 1 h. The addition of 2× sample buffer for SDS-PAGE was used to terminate the reaction. Proteins were separated on SDS-PAGE and the gels were dried. Signals were imaged using a Typhoon system (GE healthcare). Analysis of kinase activity was performed with the ImageQuant Software (GE healthcare). The ERK1-HA protein was detected by anti-HA antibody in Western blot.

### ChIP assays

The WB clone C6 cells were inoculated into encystation medium (5×10^7^ cells in 45 ml medium) and harvested after 24 h in encystation medium under drug selection and washed in phosphate-buffered saline. ChIP was performed as described previously [33] with some modifications. Formaldehyde was then added to the cells in phosphate-buffered saline at a final concentration of 1%. Cells were incubated at room temperature for 15 min and reactions were stopped by incubation in 125 mM glycine for 5 min. After phosphate-buffered saline washes, cells were lysed in luciferase lysis buffer (Promega) and protease inhibitor (Sigma) and then vortexed with glass beads. The cell lysate was sonicated on ice and then centrifuged. The chromatin extract was incubated with protein G plus/protein A-agarose (Merck) for 1 h. After removal of protein G plus/protein A-agarose, the precleared lysates were incubated with 2 µg of anti-Pax2 antibody or preimmune serum for 2 h and then incubated with protein G plus/protein A-agarose (Merck) for 1 h. The beads were washed with low salt buffer (0.1% SDS, 1% Triton X-100, 2 mM EDTA, 20 mM Tris-HCl pH 8.0, 150 mM NaCl) twice, high salt buffer (0.1% SDS, 1% Triton X-100, 2 mM EDTA, 20 mM Tris-HCl pH 8.0, 500 mM NaCl) once, LiCl buffer (0.25 M LiCl, 1% Nonidet P-40, 1% sodium deoxycholate, 1 mM EDTA, 10 mM Tris-HCl pH 8.0) once, and TE buffer (20 mM Tris-HCl, 1 mM EDTA pH 8.0) twice. The beads were resuspended in elution buffer containing 50 mM Tris-HCl, pH 8.0, 1% SDS and 10 mM EDTA at 65°C for 4 hours. To prepare DNA representing input DNA, 2.5% of precleared chromatin extract without incubation with anti-Pax2 was combined with elution buffer. Eluted DNA was purified by the QIAquick PCR purification kit (Qiagen). Purified DNA was subjected to PCR reaction followed by agarose gel electrophoresis. Primers 18S5F and 18S5R were used to amplify the *18S ribosomal RNA* gene promoter as a control for our ChIP analysis. Primers pax25F and pax25R, cwp15F and cwp15R, cwp25F and cwp25R, cwp35F and cwp35R, myb25F and myb25R, ran5F and ran5R were used to amplify *pax2*, *cwp1*, *cwp2*, *cwp3*, *myb2*, and *ran* gene promoters within the −200 to −1 region.

### Microarray analysis

RNA was quantified by A260 nm by an ND-1000 spectrophotometer (Nanodrop Technology, USA) and qualified by a Bioanalyzer 2100 (Agilent Technology) with an RNA 6000 Nano LabChip kit. RNA from the pPPax2 (or pPPax1) cell line was labeled with Cy5 and RNA from the 5′▵5N-Pac cell line was labeled with Cy3. RNA from the wild-type non-transfected WB cells cultured in growth medium was labeled with Cy5 and RNA from the wild-type non-transfected WB cells cultured in encystation medium for 24 h was labeled with Cy3. 0.5 µg of total RNA was amplified by a Low RNA Input Fluor Linear Amp kit (Agilent Technologies) and labeled with Cy3 or Cy5 (CyDye, PerkinElmer Life Sciences) during the *in vitro* transcription process. 0.825 µg of Cy-labeled cRNA was fragmented to an average size of about 50–100 nucleotides by incubation with fragmentation buffer at 60°C for 30 minutes. Correspondingly fragmented labeled cRNA was then pooled and hybridized to a *G. lamblia* oligonucleotide microarray (Agilent Technologies, USA) at 60°C for 17 h. After washing and drying by nitrogen gun blowing, microarrays were scanned with an Agilent microarray scanner (Agilent Technologies, USA) at 535 nm for Cy3 and 625 nm for Cy5. Scanned images were analyzed by Feature Extraction version 9.1 software (Agilent Technologies, USA), and image analysis and normalization software was used to quantify signal and background intensity for each feature; data were substantially normalized by the rank consistency filtering LOWESS method. All data is MIAME compliant and that the raw data has been deposited in a MIAME (http://www.mged.org/Workgroups/MIAME/miame.html) compliant database (GEO) with accession number GSE30875.

## Results

### Identification and expression of *pax2* gene

**Figure 1:**
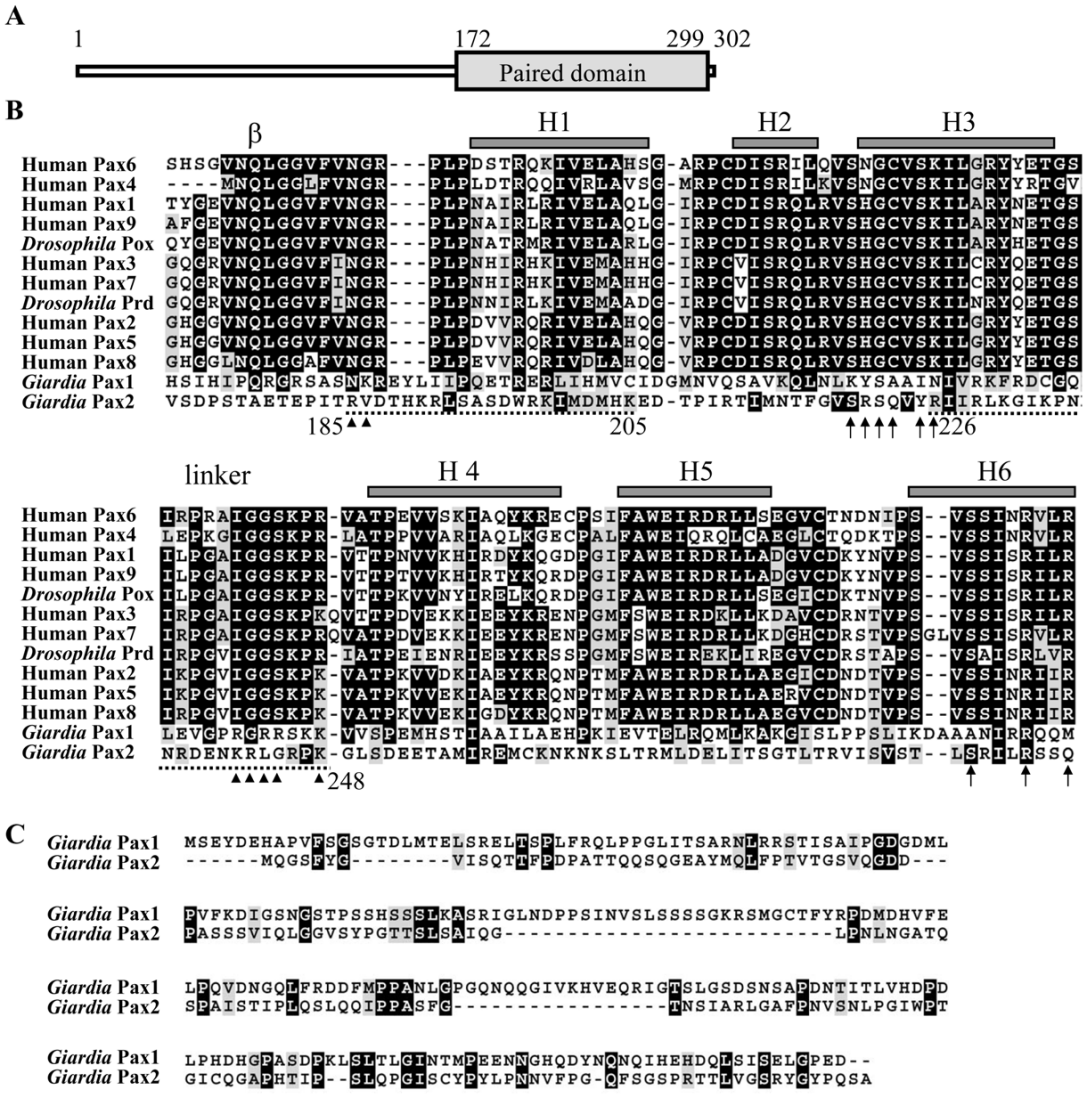
Domain architecture of Pax2 protein and alignment of the paired domains. (A) Schematic representation of the giardial Pax2 protein. The gray boxes indicate the paired domains. (B) Alignment of the paired domains. The paired domains from members of the human and *Drosophila* Pax family are analyzed by ClustalW 1.83 [80]. GenBank accession numbers for human Pax1 to 9 and *Drosophila* Prd are NM_006192, NM_000278, NM_181458, NM_006193, NM_016734, NM_000280, NM_001135254, NM_003466, NM_006194, and NM_164990, respectively. The open reading frame numbers (GenBank accession numbers) for the giardial Pax1 and Pax2 are 32686 (XM_001704983.1) and 16640 (XM_001709076.1) in the *G. lamblia* genome data base, respectively. Letters in black boxes, letters in gray boxes, and hyphens indicate identical amino acids, similar amino acids and gaps in the respective proteins, respectively. Gray boxes indicate the α helices in the paired domain of human Pax6 [58]. The arrows indicate the key residues contacting the major groove in human Pax6 or *Drosophila* Prd [58], [62]. The arrowheads indicate the residues that make contact with the minor groove/phosphodiester backbone in human Pax6 or *Drosophila* Prd [58], [62]. Two regions (residues 185–205 and 226–248) rich in basic amino acid residues are underlined by dotted lines. (C) Alignment of N-terminal regions of giardial Pax2 and Pax1 ClustalW 1.83 [80]. Letters in black boxes, letters in gray boxes, and hyphens indicate identical amino acids, similar amino acids and gaps in the respective proteins, respectively.

Two putative *pax* genes were found in the *G. lamblia* genome database (http://www.giardiadb.org/giardiadb/) [21], [53]. The *pax1* gene has been reported recently [34]. We further focused on understanding the role of Pax2 in *Giardia*. The deduced giardial Pax2 protein contains 302 amino acids with a predicted molecular mass of ∼32.71 kDa and a pI of ∼9.56. In contrast to the human Pax family, the paired domains of which are N-terminal [42], the giardial Pax2 paired domain is near the C terminus (residues 172–299) (Figure 1A). Unlike some human Pax family members, which contain a homeodomain or octapeptide, giardial Pax2 does not have these motifs as predicted by pfam (http://pfam.sanger.ac.uk/search) (Figure 1A).

The structure of the paired domain includes two subdomains, PAI and RED, each of which possesses a helix-turn-helix structure (H1–H3 for PAI and H4–H6 for RED) (Figure 1B) [43]. Structural studies of the paired domain of human Pax6 show that the H3 and H6 of each helix-turn-helix motif recognize distinct half sites and contact the DNA major grooves of these sites (Figure 1B, arrows) [58]. A β hairpin and a linker which connects two subdomains contact the DNA minor groove (Figure 1B, arrowheads) [58]. The predicted secondary structure of the Pax2 paired domain suggests similar helix-turn-helix (HTH) structure (PHD prediction, http://npsa-pbil.ibcp.fr/NPSA/npsa_references.html#phd) [59] (data not shown) to the paired domain of human pax6 [58]. As aligned in Figure 1B, the sequence of the paired domain of the giardial Pax2 has some similarity to those of the human Pax family. The paired domain of Pax2 has 13.18% sequence identity and 29.46% sequence similarity to that of human Pax6 [58]. Few of the key contact residues identified by structural studies of human Pax6 or *Drosophila* Prd are conserved in Pax2 (Figure 1B). The similarity between the giardial Pax2 and the human Pax family is limited to their paired domains (data not shown).

The paired domains of both giardial Pax2 and Pax1 are near C terminus (Figure 1A) [34]. The paired domains of Pax2 and Pax1 have 11.45% sequence identity and 28.24% sequence similarity. We further aligned the N-terminal regions of giardial Pax2 and Pax1 (Figure 1C). The N-terminal regions of giardial Pax2 and Pax1 have 14.53% sequence identity and 27.78% sequence similarity. Interestingly, eleven prolines and five serines/threonines are positionally conserved in the N-terminal regions of Pax2 and Pax1 (Figure 1C).

### Encystation-induced expression of the *pax2* gene

**Figure 2:**
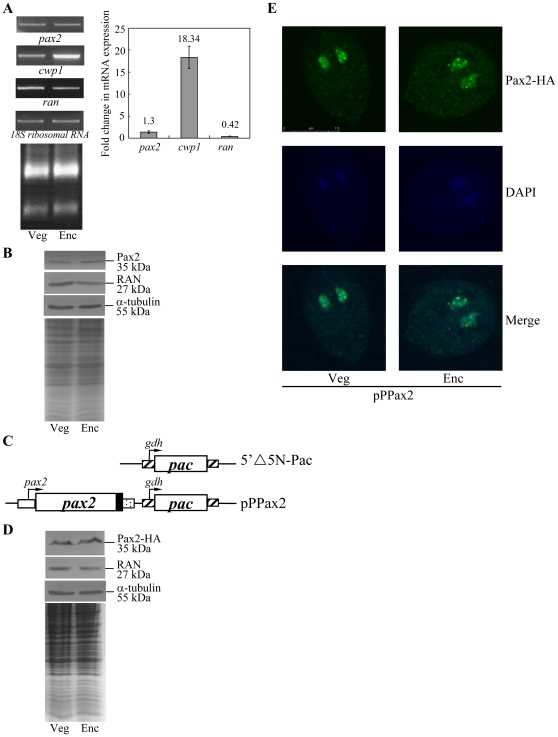
Analysis of *pax2* gene expression. (A) RT-PCR and quantitative real-time PCR analysis of *pax2* gene expression. RNA samples were prepared from *G. lamblia* wild-type non-transfected WB cells cultured in growth (Veg, vegetative growth) or encystation medium and harvested at 24 h (Enc, encystation). RT-PCR was performed using primers specific for *pax2*, *cwp1*, *ran*, and *18S ribosomal RNA* genes. Ribosomal RNA quality and loading controls are shown in the bottom panel. Representative results are shown on the left. Real-time PCR was performed using primers specific for *pax2*, *cwp1*, *ran* and *18S ribosomal RNA* genes. Transcript levels were normalized to 18S ribosomal RNA levels. Fold changes in mRNA expression are shown as the ratio of transcript levels in encysting cells relative to vegetative cells. Results are expressed as the means ± standard error of at least three separate experiments (right panel). (B) Pax2 protein levels in different stages. The wild-type non-transfected WB cells were cultured in growth (Veg, vegetative growth) or encystation medium for 24 h (Enc, encystation) and then subjected to SDS-PAGE and Western blot. The blot was probed by anti-Pax2, anti-RAN, or anti-α tubulin antibody. Representative results are shown. Equal amounts of protein loading were confirmed by SDS-PAGE and Coomassie blue staining. (C) Diagrams of the 5′▵5N-Pac and pPPax2 plasmid. The *pac* gene (open box) is under the control of the 5′- and 3′-flanking regions of the *gdh* gene (striated box). In construct pPPax2, the *pax2* gene is under the control of its own 5′-flanking region (open box) and the 3′-flanking region of the *ran* gene (dotted box). The filled black box indicates the coding sequence of the HA epitope tag. (D) Pax2 protein levels in pPPax2 stable transfectants. The pPPax2 stable transfectants were cultured in growth (Veg, vegetative growth) or encystation medium for 24 h (Enc, encystation) and then subjected to SDS-PAGE and Western blot. HA-tagged Pax2 protein was detected using an anti-HA antibody by Western blot analysis. The blot was also probed by anti-RAN or anti-α tubulin antibody. Equal amounts of protein loading were confirmed by SDS-PAGE and Coomassie blue staining. (E) Nuclear localization of Pax2. The pPPax2 stable transfectants were cultured in growth (Veg, left panels) or encystation medium for 24 h (Enc, right panels), and then subjected to immunofluorescence analysis using anti-HA antibody for detection. The product of pPPax2 localizes to the nuclei in both vegetative and encysting trophozoites (upper panels). The middle panels show the DAPI staining of cell nuclei. The bottom panels are the merged images of the DAPI staining and images of Pax2-HA.

RT-PCR and quantitative real-time PCR analysis of total RNA showed that the *pax2* transcript was present at relatively constant levels in vegetative cells and 24-h encysting cells (Figure 2A). As controls, we found that the mRNA levels of the *cwp1* and *ran* genes increased and decreased significantly during encystation, respectively (Figure 2A). The products of the *cwp1* and *ran* genes are the component of the cyst wall and the *ras*-related nuclear protein [12], [60]. To determine the expression of Pax2 protein, we generated an antibody specific to the full-length Pax2. Western blot analysis confirmed that this antibody recognized Pax2 at a size of ∼35 kDa (Figure 2B), which was almost matched to the predicted molecular mass of Pax2 (∼32.7 kDa). Pax2 was expressed at relatively constant levels during vegetative growth and encystation (Figure 2B). As a control, the levels of the giardial RAN protein (∼27 kDa) decreased significantly during encystation (Figure 2B). The levels of the α tubulin protein (∼55 kDa) were relatively constant during vegetative growth and encystation (Figure 2B) [56].

### Localization of the Pax2 protein

To determine the role of Pax2 protein, we prepared construct pPPax2 in which the *pax2* gene is controlled by its own promoter and contains an HA epitope tag at its C terminus (Figure 2C) and stably transfected it into *Giardia*. Similar to the expression pattern of the endogenous Pax2 protein, Pax2-HA was expressed at relatively constant levels during vegetative growth and encystation (Figure 2D). The HA-tagged Pax2 was detected in the nuclei during vegetative growth and encystation (Figure 2E), indicating that Pax2 is a nuclear protein in *Giardia*. As a negative control, there was no staining for anti-HA antibody detection in the 5′▵5N-Pac cell line, which expressed only the puromycin selection marker (Figure 2C; data not shown).

**Figure 3:**
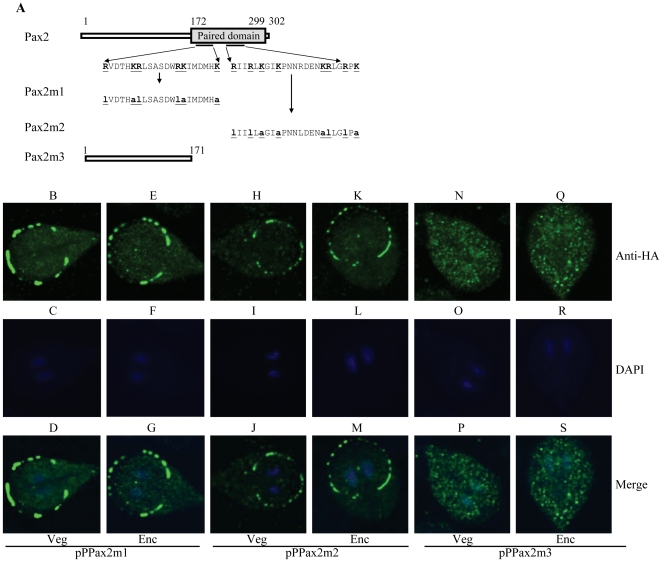
Localization of Pax2 mutants. (A) Diagrams of the Pax2 and Pax2m1-3 proteins. The gray box indicates the paired domain. Pax2m3 does not contain the C-terminal paired domain and C-terminal region (residues 172–302). Pax2m1 and Pax2m2 contain a mutation of two stretches of basic amino acids located inside of the paired domain (residues 185–205 for Pax2m1; residues 226–248 for Pax2m2). The *pax2* gene was mutated and subcloned to replace the wild type *pax2* gene in the backbone of pPPax2 (Figure 2C), and the resulting plasmids pPPax2m1-3 were transfected into *Giardia*. (B) Immunofluorescence analysis of Pax2m1-3 distribution. The pPPax2m1-3 stable transfectants were cultured in growth (Veg, vegetative growth) or encystation medium for 24 h (Enc, encystation) and then subjected to immunofluorescence analysis using anti-HA antibody for detection.

We further identified the portion of Pax2 that is sufficient to direct the protein to the nuclei. No typical nuclear localization signal was predicted using the PSORT program (http://psort.nibb.ac.jp/) [61]. Two regions rich in basic amino acid residues may be putative nuclear localization signals (residues 185–205 and 226–248) (Figure 1B, dotted line). These two putative nuclear localization signals are located inside the paired domain (Figure 1B). Mutation of the basic amino acids between residues 185–205 and 226–248 (pPPax2m1 and pPPax2m2) (Figure 1B and Figure 3A) resulted in a significant loss of nuclear localization in both vegetative and encysting cells (Figure 3B–M), suggesting that these basic residues may play an important role in the exclusively nuclear localization. The staining was distributed in sucking disk and some small vesicles in cytosol (Figure 3H–S). Nuclear staining was faint, but detectable (Figure 3B–M). Deletion of the C-terminal region containing the paired domain and the C-terminal 3 amino acids (residues 172–302, pPPax2m3, Figure 1A and Figure 3A) resulted in a significant decrease of nuclear localization (Figure 3N–S). The staining was evenly distributed in both the nuclei and some small vesicles in cytosol of both vegetative and encysting cells (Figure 3N–S).

### Identification of the Pax2 binding sites

**Figure 4:**
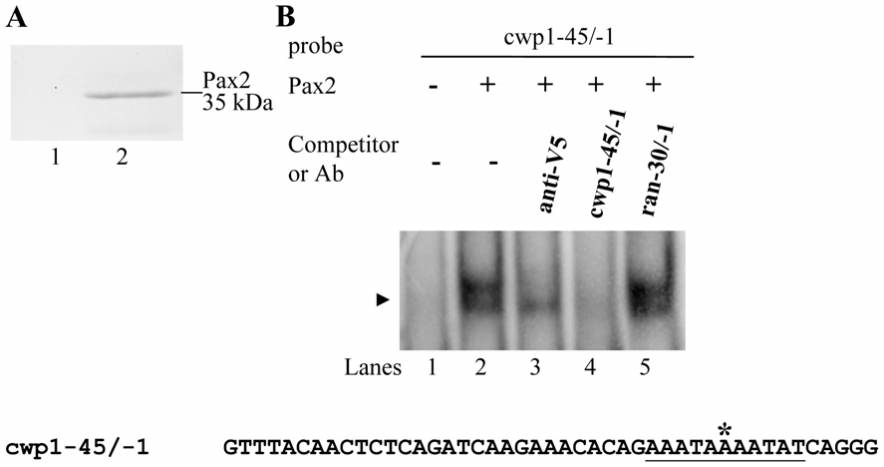
DNA-binding ability of Pax2 revealed by electrophoretic mobility shift assays. (A) Western blot analysis of recombinant Pax2 protein with a V5 tag at its C terminus purified by affinity chromatography. The purified Pax2 protein is detected by anti-V5-HRP antibody (lane 2). *E. coli* lysate with the vector only (pET101/D-TOPO) was used as a negative control and no detection was observed for this control with anti-V5-HRP antibody (lane 1). (B) Detection of Pax2 binding sites. Electrophoretic mobility shift assays were performed using purified Pax2 and the ^32^P-end-lableled oligonucleotide probe cwp1-45/−1 (−45 to −1 relative to the translation start site of the *cwp1* gene). Components in the binding reaction mixtures are indicated above the lanes. The Pax2 binding specificity for the cwp1-45/−1 probe was confirmed by competition and supershift assays. Some reaction mixtures contained 200-fold molar excess of cold oligonucleotides cwp1-45/−1 or ran-30/−1 or 0.8 µg of anti-V5 antibody, as indicated above the lanes. The arrowhead indicates the shifted complex. The transcription start site of the *cwp1* gene determined from 24-h encysting cells is indicated by an asterisk [13]. The AT-rich Inr element spanning the transcription start site is underlined.

The nuclear localization of Pax2 suggested that it might also function as a transcription factor in *G. lamblia*. To test its DNA-binding activity, we expressed Pax2 with a C-terminal V5 tag in *E. coli* and purified it to >95% homogeneity (data not shown). An anti-V5-HRP antibody specifically recognized the recombinant V5-tagged Pax2 in Western blots (Figure 4A).

To determine whether purified Pax2 binds DNA, we performed electrophoretic mobility shift assays with double-stranded DNA sequences from the 5′-flanking region of an encystation-induced gene, *cwp1* gene. Incubation of a labeled double-stranded DNA probe cwp1-45/−1 with Pax2 resulted in the formation of shifted bands (Figure 4B, lane 2). cwp1-45/−1 is the region from −45 to −1 bp relative to the translation start site of the *cwp1* gene. Pax2 did not bind to either single strand of the cwp1-45/−1 probe (data not shown). The binding specificity was confirmed by competition and supershift assays (Figure 4B, lanes 3–5). The intensity of the shifted cwp1-45/−1 band was decreased significantly by the addition of anti-V5 antibody (Figure 4B, lane 3). The formation of the shifted cwp1-45/−1 bands was almost totally competed by a 200-fold molar excess of unlabeled cwp1-45/−1 but not by the same excess of a nonspecific competitor, cwp1-45/-1m7 (Figure 4B, lanes 4 and 5).

**Figure 5:**
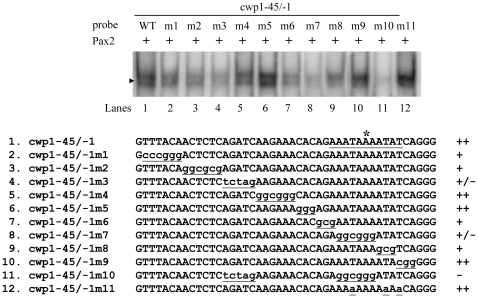
Mutation analysis of the cwp1-45/−1 probe sequence containing the putative Pax2 binding site. Electrophoretic mobility shift assays were performed using purified Pax2 and various ^32^P end-labeled cwp1-45/−1 mutant probes as described. Base changes in the mutants are shown in underlined lowercase type. Components in the binding reaction mixtures are indicated above the lanes. The arrowhead indicates the shifted complex. The transcription start site of the *cwp1* gene determined from 24-h encysting cells is indicated by an asterisk [13]. The AT-rich Inr element spanning the transcription start site is underlined. “+”, “+/−”, and “−” represent moderate binding, weak binding, and no binding, respectively. “+++” and “++” represent strong binding.

Scanning mutagenesis of the cwp1-45/−1 probe showed that substitutions within the AGATC or AATAAA sequence significantly decreased the DNA-protein interaction (cwp1-45/-1m3 and 7)(Figure 5, lanes 4 and 8), but mutations of the other regions caused a minor decrease in binding (cwp1-45/-1m1, 2, 6, and 8)(Figure 5, lanes 2, 3, 7, and 9). Mutations of some other regions did not change the binding (cwp1-45/-1m4, 5, and 9)(Figure 5, lanes 5, 6, and 10). Mutation of both regions almost abolished binding (cwp1-45/-1m10) (Figure 5, lane 11). Substitutions of the three Ts within the AAATAAAATAT region did not change the binding activity (cwp1-45/-1m11) (Figure 5, lane 12).

**Figure 6:**
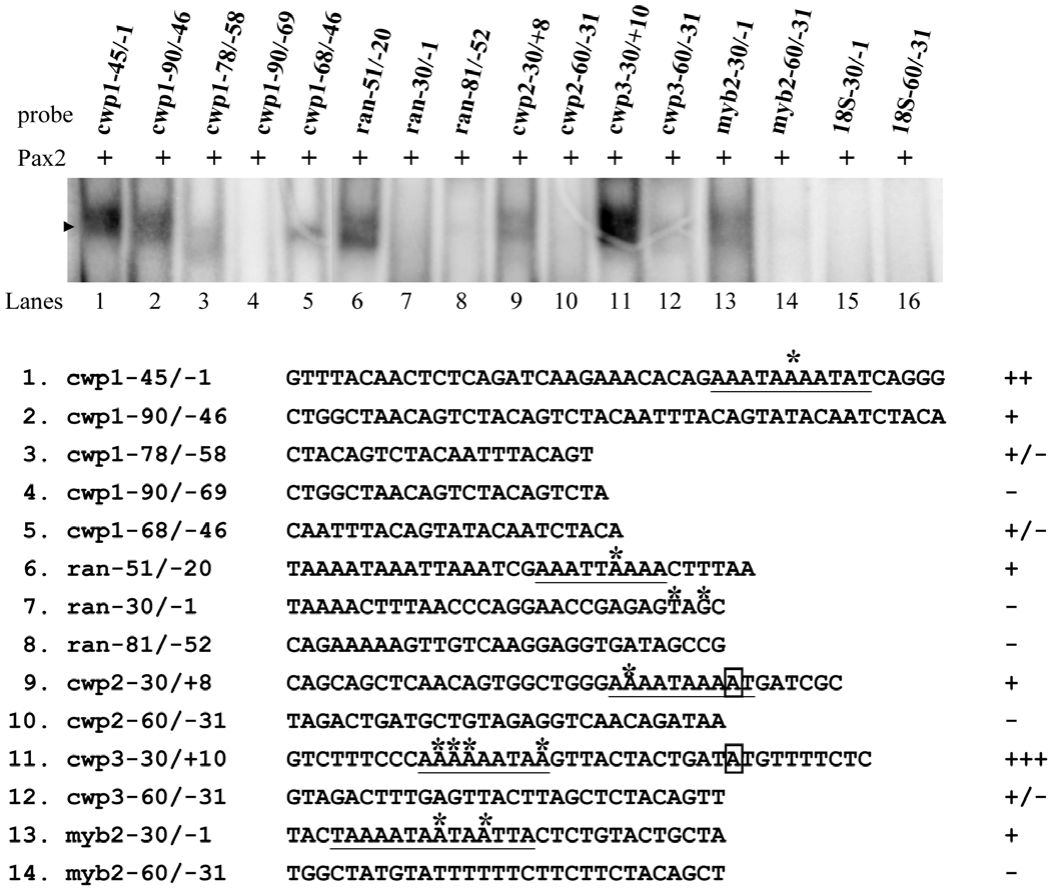
Detection of Pax2 binding sites in multiple promoters. Electrophoretic mobility shift assays were performed using purified Pax2 and various ^32^P-end-lableled oligonucleotide probes as described. Components in the binding reaction mixtures are indicated above the lanes. The arrowhead indicates the shifted complex. The transcription start sites of the *cwp2*, *cwp3*, and *myb2* genes determined from 24-h encysting cells are indicated by asterisks [12], [14]. The transcription start sites of the *ran* gene determined from vegetative cells are indicated by asterisks [26]. The AT-rich Inr elements spanning the transcription start sites are underlined. The translation start sites of the *cwp2* and *cwp3* genes are framed. “18S” represents 18S ribosomal RNA. “+”, “+/−”, and “−” represent moderate binding, weak binding, and no binding, respectively. “+++” and “++” represent strong binding.

Pax2 was also shown to bind to cwp1-90/−46, and within this region it bound weakly to the 3′-region (cwp1-68/−46) and the middle region (cwp1-78/−58), but not to the 5′-region (cwp1-90/−69) (Figure 6, lanes 2–5). Pax2 also bound to a well characterized *ran* core promoter, ran-51/−20 (Figure 6, lane 6) [26], but not to ran-30/−1 or ran-81/−52 (Figure 6, lanes 7 and 8). We also tested whether Pax2 binds to the 5′-flanking region of other encystation-induced genes, *cwp2*, *cwp3*, and *myb2*. We found that Pax2 bound strongly to the cwp3-30/+10 probe (Figure 6, lane 11) and weakly to the cwp2-30/+8, cwp3-60/−31, and myb2-30/−1 probes (Figure 6, lanes 9, 12, and 13), but it did not bind to the cwp2-60/−31 and myb2-60/−31 probes (Figure 6, lanes 10 and 14). Pax2 did not bind to the 18S-30/−1, and 18S-60/−31 probes, which do not contain AT-rich sequence (Figure 6, lanes 15 and 16). The results suggest that Pax2 can bind specifically to the *cwp1*, *cwp2*, *cwp3*, *myb2*, and *ran* AT-rich Inr regions (Figure 5 and Figure 6, underlined region).

**Figure 7:**
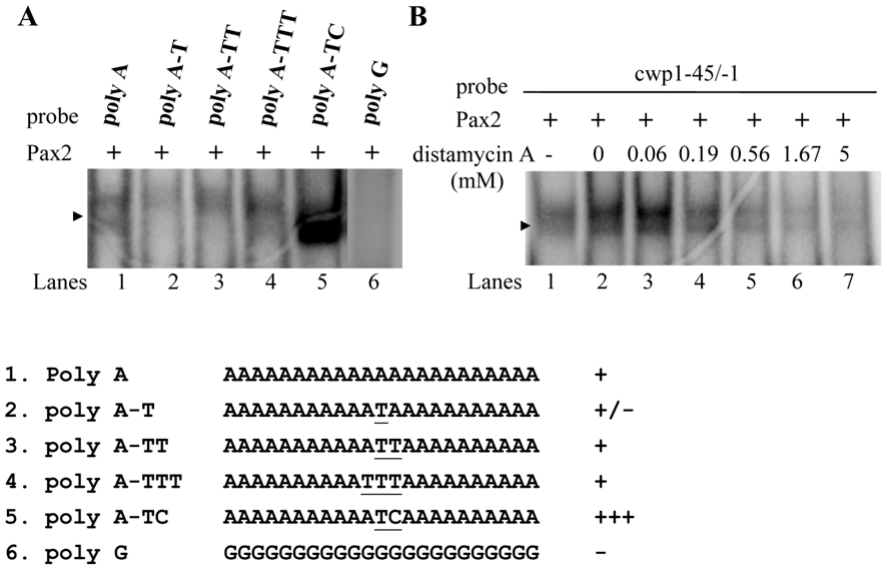
Analysis of Pax2 binding ability. (A) Pax2 may bind to AT-rich sequence. Electrophoretic mobility shift assays were performed using purified Pax2 and various ^32^P-end-lableled oligonucleotide probes as described. Components in the binding reaction mixtures are indicated above the lanes. The arrowhead indicates the shifted complex. “+”, “+/−”, and “−” represent moderate binding, weak binding, and no binding, respectively. “+++” and “++” represent strong binding. (B) Effect of distamycin A on the binding of Pax2 to DNA. ^32^P end-labeled cwp1-45/−1 probe was incubated with Pax2 in the absence (lane 1) or presence of distamycin A (lanes 3–7). The arrowhead indicates the shifted complex. DistamycinA was dissolved in Me2SO. Adding Me2SO to the reaction mix did not decrease the Pax2 binding activity (lane 2).

We also tested whether Pax2 binds to specific AT-rich sequences. Interestingly, Pax2 also bound to a poly(A) sequence and a poly (A) sequence with a T, TT, TTT, or a TC insertion (Figure 7A, lanes 1–5), but it did not bind to a poly (G) sequence (Figure 7A, lane 6), indicating that the Pax2 binding sequence contains AT-rich sequences.

Studies suggest that *Drosophila* Prd and human Pax6 can bind to both DNA major (using the H3 and H6; Figure 1B, arrows) and minor grooves (using the β hairpin and linker which connects PAI and RED subdomains; Figure 1B, arrowheads) [58], [62]. To investigate how Pax2 binds DNA, we used distamycin A, which binds to the minor groove of AT-rich DNA sequences, as a competitive inhibitor of Pax2 binding [63]. As shown in Figure 7B, the binding of Pax2 to DNA decreased with increasing concentrations of distamycin A. However, the binding was not completely inhibited at concentrations ∼5 mM, suggesting that Pax2 may bind to both DNA major and minor grooves.

The paired domains of the human Pax proteins are known to be important for DNA binding [42]. We found that a Pax2 mutant (Pax2m3) with a deletion of the C-terminal region containing the paired domain (residues 172–302, Pax2m1) reduced nuclear localization and increased cytosol localization (Figure 3A and 3N–S). To understand whether the C-terminal paired domain is also important for DNA binding, the Pax2m3 mutant was expressed in *E. coli* and purified. We found that the purified Pax2m3 did not bind to the cwp1-45/−1, cwp2-30/+8, cwp3-30/+10, and ran-51/−20 probes (Figure S1B–E), indicating that the C-terminal paired domain is important for DNA binding. Two putative nuclear localization signals have been found inside of the paired domain (residues 185–205, 226–248). Both are important for nuclear localization (Figure 3). We also tried to understand whether the regions we tested for nuclear localization are important for DNA binding. Mutation of the basic amino acids between residues 185 and 205 (Pax2m1) or between residues 226 and 248 (Pax2m2) (Figure 3A) resulted in a significant decrease of binding activity to the cwp1-45/−1, cwp2-30/+8, cwp3-30/+10, and ran-51/−20probes (Figure S1B–E). Similar or higher levels of Pax2m1-3 were added to the binding reaction mixture (Figure S1A). The results suggest that the basic residues inside the DNA binding domain may play an important role in DNA binding.

### Overexpression of Pax2 induced the expression of *cwp1-3*, and *myb2* genes

**Figure 8:**
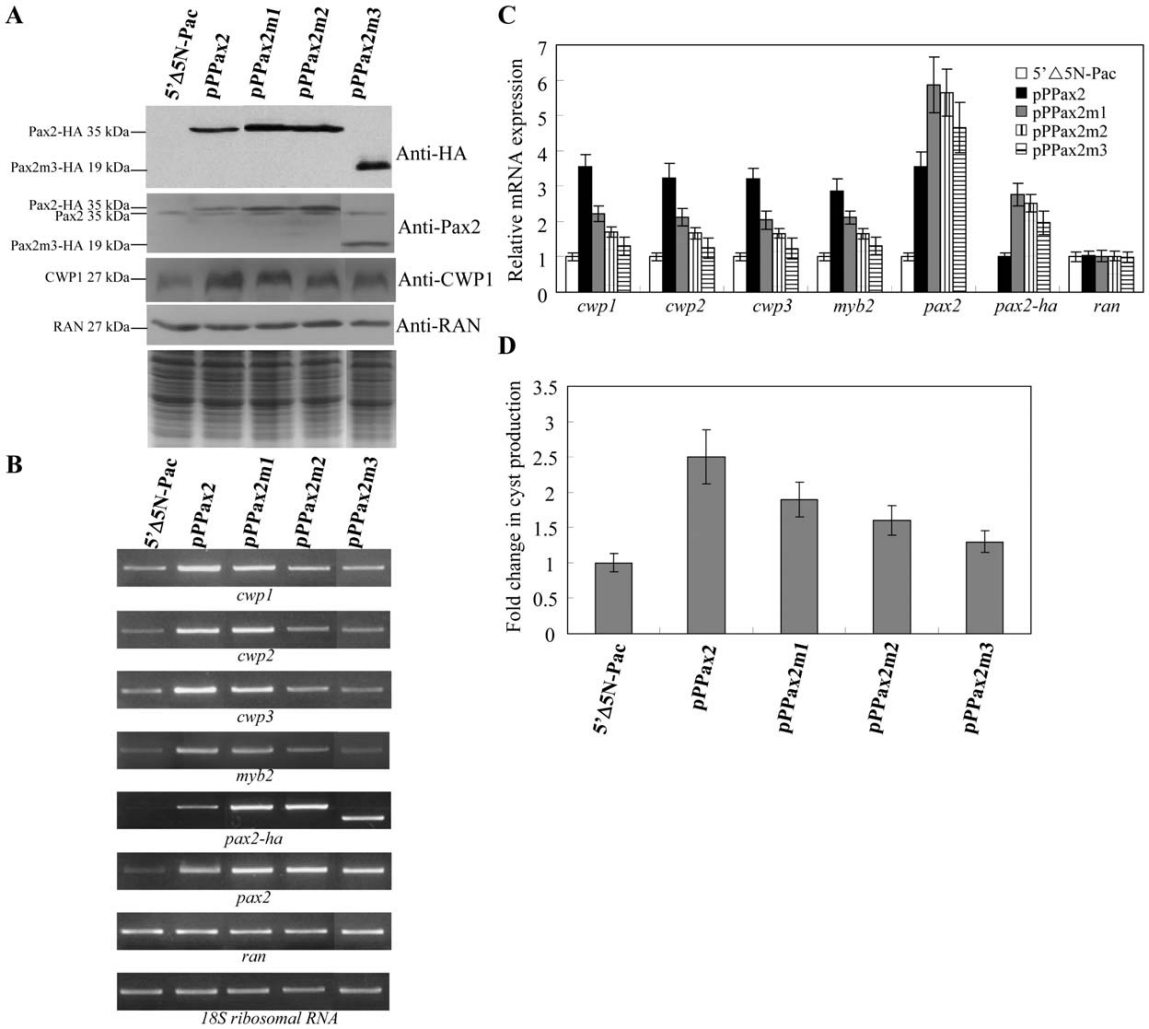
Activation of *cwp1-3* and *myb2* gene expression in the Pax2 overexpressing cell line. (A) Overexpression of Pax2 increased the levels of CWP1 protein. The 5′▵5N-Pac, pPPax2, and pPPax2m1-3 stable transfectants were cultured in growth medium and then subjected to SDS-PAGE and Western blot. The blot was probed by anti-Pax2, anti-HA and anti-CWP1 antibody. Equal amounts of protein loading were confirmed by SDS-PAGE and Coomassie blue staining. Representative results are shown. (B) RT-PCR analysis of gene expression in the Pax2 and Pax2m1-3 overexpressing cell lines. The 5′▵5N-Pac, pPPax2, and pPPax2m1-3 stable transfectants were cultured in growth medium and then subjected to RT-PCR analysis using primers specific for *pax2-ha*, *pax2*, *cwp1*, *cwp2*, *cwp3*, *myb2*, *ran*, and *18S ribosomal RNA* genes. (C) Quantitative real-time PCR analysis of gene expression in the Pax2 and Pax2m1-3 overexpressing cell lines. Real-time PCR was performed using primers specific for *pax2-ha*, *pax2*, *cwp1*, *cwp2*, *cwp3*, *myb2*, *ran*, and *18S ribosomal RNA* genes. Similar mRNA levels of the *ran* and *18S ribosomal RNA* genes for these samples were detected. Transcript levels were normalized to 18S ribosomal RNA levels. Fold changes in mRNA expression are shown as the ratio of transcript levels in the pPPax2 or pPPax2m1-3 cell line relative to the 5′▵5N-Pac cell line. Results are expressed as the means ± standard error of at least three separate experiments. (D) Cyst count. The 5′▵5N-Pac, 5′▵5N-Pac, pPPax2, and pPPax2m1-3 stable transfectants were cultured in growth medium and then subjected to cyst count as described under “**Methods**”. The sum of total cysts is expressed as relative expression level over control. Values are shown as means ± standard error.

To study the role of Pax2 in *G. lamblia*, we expressed *pax2* by its own promoter (pPPax2; Figure 2C) and observed its gene expression. A ∼35-kDa protein was detected (Figure 8A), which is matched to the predicted molecular mass of Pax2 (∼32.7 kDa) with the HA tag (∼0.8 kDa). Overexpression of Pax2 in the pPPax2 cell line can be confirmed by the anti-Pax2 antibody. The size of the overexpressed Pax2-HA is slightly larger than that of the endogenous Pax2 (Figure 8A). We found that Pax2 overexpression resulted in a significant increase of the CWP1 protein levels during vegetative growth (Figure 8A). As a control, similar levels of intensity of the giardial RAN protein (∼27 kDa) were detected by anti-RAN antibody (Figure 8A). RT-PCR and quantitative real-time PCR analysis showed that the mRNA levels of the endogenous *pax2* plus vector-expressed *pax2* in the Pax2-overexpressing cell line increased by ∼3.5-fold (*p*<0.05) (Figure 8, B and C) relative to the control cell line, which expressed only the puromycin selection marker (5′▵5N-Pac) (Figure 2C) [54]. The mRNA levels of the endogenous *cwp1*, *cwp2*, *cwp3*, and *myb2* genes in the Pax2 overexpressing cell line increased by ∼2.9 to 3.5-fold (P<0.05) relative to the control cell line (Figure 8B and C). Similar mRNA levels of the *ran* and *18S ribosomal RNA* genes were detected (Figure 8B and C). We further investigated the effect of giardial Pax2 on cyst formation. In previous studies, we have found that some *G. lamblia* trophozoites may undergo spontaneous differentiation [51]. We obtained consistent cyst count data for vegetative *G. lamblia* cultures during growth to stationary phase (∼4800 cysts/ml for 5′Δ5N-Pac cell line) [51]. In this study, we found that the cyst number in the Pax2 overexpressing cell line increased by ∼2.5-fold (P<0.05) relative to the control cell line, which expresses only the puromycin selection marker (5′Δ5N-Pac) (Figure 2C), indicating that the overexpressed Pax2 can increase the cyst formation (Figure S1D). Similar results were obtained during encystation (data not shown). The results suggest that the overexpressed Pax2 can transactivate the *cwp1*, *cwp2*, *cwp3*, and *myb2* genes.

To further understand the function of giardial Pax2, we observed the effect of overexpression of the Pax2m1, Pax2m2, and Pax2m3 mutants that have lower DNA binding activity (Figure S1). The mutations resulted in a significant loss of nuclear localization of Pax2m1-3 (Figure 3). We found that the levels of Pax2m1, Pax2m2, or Pax2m3 protein increased significantly compared with that of wild type Pax2 during vegetative growth in both anti-HA and anti-Pax2 Western blots (Figure 8A). We further analyzed whether the transcript levels of the Pax2m1, Pax2m2, or Pax2m3 were changed. As shown by RT-PCR and quantitative real-time PCR analysis, the levels of HA-tagged *pax2m1*, *pax2m2*, and *pax2m3* mRNA increased by ∼2.76, ∼2.52, or ∼1.96-fold (p<0.05) compared with that of wild type HA-tagged *pax*2 during vegetative growth (Figure 8B and C). This suggests a negative autoregulation of the *pax2* gene. We did not detect any HA-tagged pax2 transcripts in the 5′▵5N-Pac control cell line (Figure 8B). We also found that the levels of the CWP1 protein and the *cwp1*, *cwp2*, *cwp3*, and *myb2* mRNA and cyst formation decreased significantly in the Pax2m2 or Pax2m3 overexpressing cell line relative to the wild-type Pax2 overexpressing cell line (Figure 8, B, C, and D). Similar results were obtained during encystation (data not shown). The results suggest a decrease of transactivation activity of Pax2m2 and Pax2m3. The transactivation activity of the Pax2m1 mutant only decreased slightly (Figure 8A, B, C, and D), possibly because of its partial loss of DNA binding activity, partial mis-localization and its relative higher expression as compared with other Pax2 mutants (Figure 8B and C). Therefore, it is possible that the Pax2m1 mutant still possesses partial transactivation activity.

**Figure 9:**
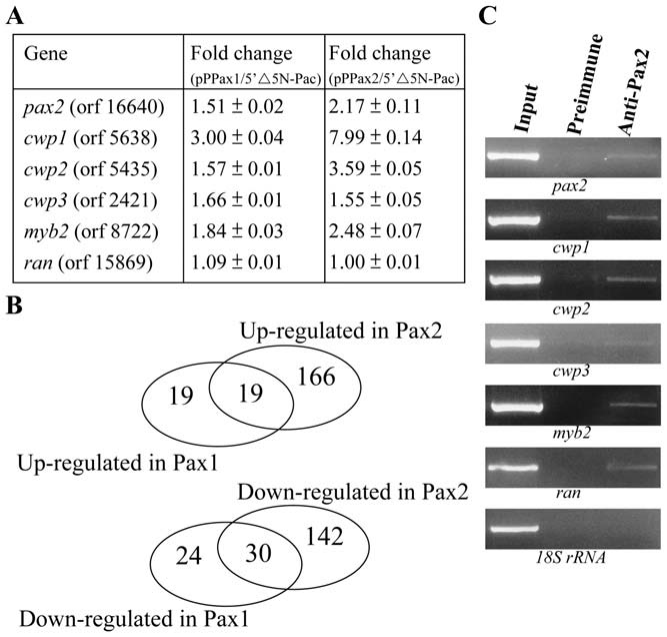
Recruitment of Pax2 to the *cwp1-3* and *myb2* promoters. (A) Microarray analysis. Microarray data were obtained from the 5′▵5N-Pac and pPPax1 (or pPPax2) cell lines during vegetative growth. Fold changes are shown as the ratio of transcript levels in the pPPax1 (or pPPax2) cell line relative to the 5′▵5N-Pac cell line. Results are expressed as the means ± standard error of at least three separate experiments. (B) Pax2 and Pax1 overexpression generated similar gene expression patterns. The Venn diagrams illustrate the overlap of altered gene expression between the Pax2 and Pax1 overexpressing cells. Thirty eight and 185 genes were up-regulated (i.e. increased levels of gene expression relative to the control) in the Pax1 and Pax2 overexpressing cells, respectively. Among them, nineteen genes overlap. Fifty four and 172 genes were down-regulated in the Pax1 and Pax2 overexpressing cells, respectively. Among them, thirty genes overlap. (C) ChIP assays. The non-transfected WB cells were cultured in growth medium for 24 h and then subjected to ChIP assays. Anti-Pax2 antibody was used to assess binding of Pax2 to endogenous gene promoters. Preimmune serum was used as a negative control. Immunoprecipitated chromatin was analyzed by PCR using primers that amplify the 5′-flanking region of specific genes. At least three independent experiments were performed. Representative results are shown. Immunoprecipitated products of Pax2 yielded more PCR products of *pax2*, *cwp1*, *cwp2*, *cwp3*, *myb2*, and *ran* promoters, indicating that Pax2 was bound to these promoters. The *18S ribosomal RNA* gene promoter was used as a negative control for our ChIP analysis.

Oligonulceotide microarray assays confirmed the up-regulation of the *cwp1*, *cwp2*, *cwp3*, and *myb2* gene expression in the Pax2-overexpressing cell line to ∼1.55- to ∼7.99-fold of the levels in the control cell line (Figure 9A). Similar mRNA levels of the *ran* gene were detected (Figure 9A). It has been found that the *cwp1*, *cwp2*, *cwp3*, and *myb2* gene expression increased in the Pax1-overexpressing cell line to ∼1.57- to ∼3.00-fold (Figure 9A) [34]. We found that 185 and 172 genes were significantly up-regulated (>2 fold) and down-regulated (<1/2) (*p*<0.05) in the Pax2 overexpressing cells relative to the vector control (Figure 9B and Table S2). We also compared gene expression profiles of the Pax1 overexpressing cells and found that 38 and 54 genes were significantly up-regulated (>2 fold) and down-regulated (<1/2) (*p*<0.05) in the Pax1 overexpressing cells relative to the vector control (Figure 9B and Table S3). Interestingly, nineteen genes were up-regulated in both the Pax2 and Pax1 overexpressing cells (Figure 9B and Table S4). Thirty genes were down-regulated in both the Pax2 and Pax1 overexpressing cells (Figure 9B and Table S4). Only one gene is up-regulated in the Pax2 overexpressing cells but down-regulated in the Pax1 overexpressing cells (Orf 3731, Hypothetical protein). The expression levels of the *pax2* or *pax1* gene in the Pax2 or Pax1 overexpressing cell line increased by ∼2.17 or 1.51-fold (P<0.05) (Figure 9A) [34]. The results suggest that the profiles of gene expression in the Pax2 and Pax1 overexpressing cells significantly overlap in the same direction.

### Recruitment of Pax2 to the *pax2*, *cwp1*, *cwp2*, *cwp3*, and *myb2* promoters

We further used ChIP assays to study the association of Pax2 with specific promoters in the Pax2 overexpressing cell line. We found that Pax2 was associated with its own promoter and the *cwp1*, *cwp2*, *cwp3*, *myb2*, and *ran* gene promoters during vegetative growth or during encystation (Figure 9C and data not shown). However, Pax2 was not associated with the *18S ribosomal RNA* gene promoter, which has no Pax2 binding site in the <200 bp 5′-flanking region (Figure 9C).

### Phosphorylation of Pax2 and Pax1 by ERK1 associated complexes

**Figure 10:**
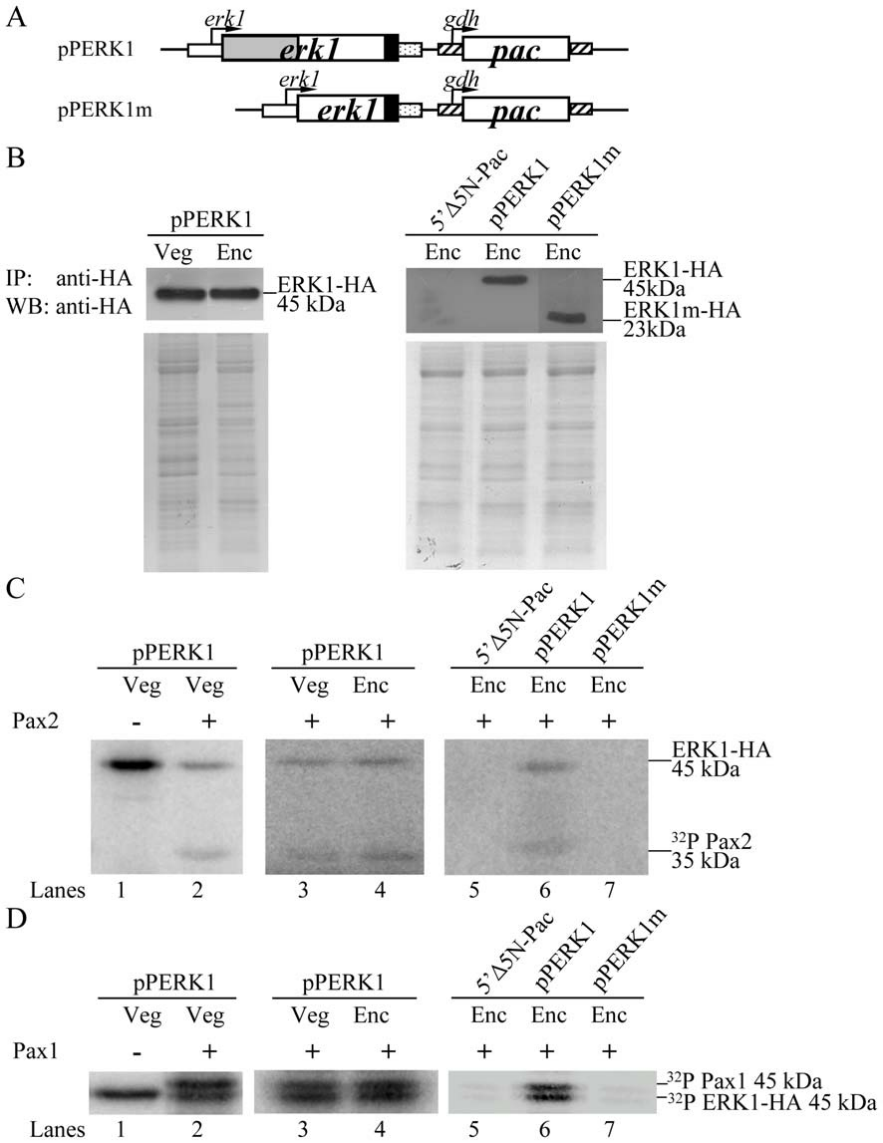
Phosphorylation of Pax2 and Pax1 proteins by ERK1 associated complexes. (A) Diagrams of the pPERK1 and pPERK1m plasmids. The *pac* gene (open box) expression cassette is the same as in Figure 2C. The *erk1* gene is under the control of its own 5′-flanking region (open boxes) and the 3′-flanking region of the *ran* gene (dotted box). ERK1m does not contain the predicted kinase domain (residues 26–202)(gray box). The filled box indicates the coding sequence of the HA epitope tag. (B) Similar levels of immunoprecipitated ERK1 protein from the vegetative and encysting pPERK1 cultures used in kinase assays. The pPERK1 stable transfectants were cultured in growth (Veg, vegetative growth) or encystation medium for 24 h (Enc, encystation) and then subjected to IP-kinase assays using anti-HA antibody. The addition of similar levels of the HA-tagged ERK1 protein from the vegetative and encysting pPERK1 cultures in each kinase reaction was confirmed by Western blot using an anti-HA antibody (left panel). The addition of similar levels of the HA-tagged ERK1 protein from the encysting pPERK1, and pPERK1m cultures in each kinase reaction was confirmed by Western blot using an anti-HA antibody (right panel). Equal amounts of protein loading were confirmed by SDS-PAGE and Coomassie blue staining. (C) Encystation-induced kinase activity of ERK1 for Pax2 substrate. The pPERK1 stable transfectants were cultured in growth (Veg, vegetative growth) or encystation medium for 24 h (Enc, encystation) and then subjected to IP-kinase assays using anti-HA antibody. Kinase activity was measured using purified recombinant Pax2 as a substrate. As a negative control, an IP-kinase assay was performed with the encysting 5′▵5N-Pac cultures which did not express the HA-tagged ERK1 protein (lane 5). Another IP-kinase assay was performed with the encysting pPERK1m cultures which expressed the ERK1m-HA protein without the predicted kinase domain (residues 26–202) (lane 7). To account for ERK1 autophosphorylation, an additional control without substrate but with immunoprecipitated ERK1-HA was also included (lane 1). (D) Encystation-induced kinase activity of ERK1 for Pax1 substrate. IP-kinase assays were performed as described above, except that Pax1 substrate was used.

In previous studies, a member of the MAPK family, ERK1, has been identified to exhibit an increased kinase activity during early encystation [18], [49]. To understand whether giardial Pax2 and Pax1 are regulated by the MAPK/ERK1 pathway, we prepared construct pPERK1 in which the *erk1* gene is controlled by its own promoter with an HA tag fused at its C terminus (Figure 10A) and stably transfected it into *G. lamblia* [18]. The ERK1-HA protein levels increased during encystation [18]. We found that overexpression of ERK1 resulted in increased levels of the CWP1 proteins and cyst formation [18]. We also found a significant increase of the *cwp1*, *cwp2*, or *myb2* mRNA levels in the ERK1-overexpressing cell line relative to the vector control cell line [18]. The kinase activity of ERK1-HA in the pPERK1 cell line was determined by IP kinase assays using anti-HA antibody. We used purified Pax2 and Pax1 as substrates for kinase assays. We found that the ERK1-HA associated complexes can phosphorylate both the Pax2 and Pax1 proteins (Figure 10C and D). Interestingly, the kinase activity for both substrates increased during encystation (Figure 10C and D). A 2-fold and 1.8-fold increase was found in the Pax2 and Pax1 reaction, respectively (data not shown). Note that the ERK1-HA protein levels increased during encystation [18], but similar levels of the immunoprecipitated ERK1-HA from the vegetative and encysting pPERK1 cultures were used in the IP-kinase assays as confirmed by Western blot using anti-HA antibody (Figure 10B). As a negative control, the ERK1-HA associated kinase activity was not detected in the 5′▵5N-Pac cell line, which did not express the ERK1-HA protein (Figure 2C and Figure 10C and D). The results indicate that Pax2 and Pax1 may be a target of ERK1 pathway and the function of Pax2 and Pax1 is coincided with the increased ERK1 activity during encystation. The results suggest that ERK1 pathway may regulate *Giardia* differentiation into cysts through the regulation of Pax2 and Pax1 phosphorylation.

**Figure 11:**
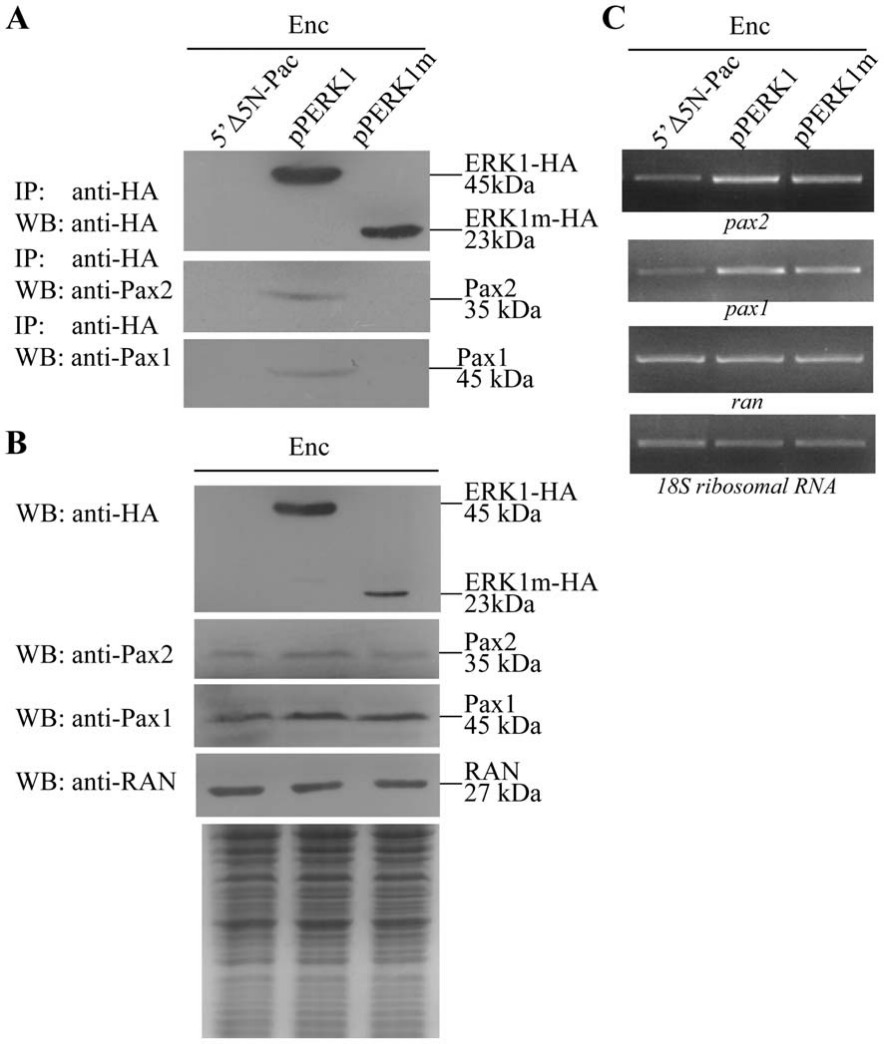
Interaction between ERK1 and Pax2 (or Pax1). (A) Co-immunoprecipitation assay. The 5′Δ5N-Pac, pPERK1, and pPERK1m stable transfectants were cultured in encystation medium for 24 h. Proteins from cell lysates were immunoprecipitated using anti-HA antibody conjugated to beads. The precipitates were analyzed by Western blot with anti-HA, anti-Pax2, or anti-Pax1 antibody as indicated. (B) Expression of HA tagged ERK1, Pax2, and Pax1 proteins in whole cell extracts. The 5′Δ5N-Pac, pPERK1, and pPERK1m stable transfectants were cultured in encystation medium for 24 h (Enc, encystation) and then subjected to Western blot analysis. The blot was probed by anti-HA, anti-Pax2, anti-Pax1, and anti-RAN antibody. Equal amounts of protein loading were confirmed by SDS-PAGE and Coomassie blue staining. (C) RT-PCR analysis of gene expression in the ERK1- and ERK1m-overexpressing cell line. The 5′Δ5N-Pac, pPERK1, and pPERK1m stable transfectants were cultured in encystation medium for 24 h (Enc, encystation) and then subjected to RT-PCR analysis. PCR was performed using primers specific for *pax2*, *pax1*, *ran*, and *18S ribosomal RNA* genes.

To further understand whether giardial Pax2 or Pax1 is regulated by the MAPK/ERK1 pathway, we constructed a pPERK1m plasmid that encodes a mutant ERK1 (ERK1m) lacking the predicted kinase domain (residues 26–202) (Figure 11A) [18]. We found that deletion of the predicted kinase domain resulted in decreased levels of the CWP1 proteins and cyst formation [18]. We also found a significant decrease of the *cwp1*, *cwp2*, or *myb2* mRNA levels in the ERK1m-overexpressing cell line relative to the wild-type ERK1 overexpressing cell line [18]. We further performed another IP-kinase assay with the encysting pPERK1m cultures. Similar levels of the immunoprecipitated ERK1-HA or ERK1m-HA were used in the IP-kinase assays as confirmed by Western blot using anti-HA antibody (Figure 10B). No kinase activity was detected in the ERK1m-HA associated lysates using Pax2 or Pax1 as a substrate (Figure 10C and D), suggesting that ERK1m had no kinase activity.

ERK can be autophosphorylated at regulatory Tyr/Thr residues and it has to be maintained at low levels to prevent its autoactivation [64]. We also found that the giardial ERK1 can be autophosphorylated using a control without substrate addition in the kinase assays with immunoprecipitated ERK1-HA (Figure 10C and D).

### Interaction between ERK1 and Pax2 (or Pax1)

It is possible that ERK1 may interact with Pax2 or Pax1 transcription factors. We then performed co-immunoprecipitation experiments in ERK1 overexpressing cell line. We lysed the cells and immunoprecipitated HA-tagged ERK1 with anti-HA antibody. Western blots of immunoprecipitates probed with anti-HA, anti-Pax2, and anti-Pax1 indicate that ERK1-HA co-precipitates with Pax2 or Pax1 (Figure 11A). As a control, the anti-HA antibody did not immunoprecipitate ERK1 and Pax2 (or Pax1) in the control cell line, which expressed only the puromycin selection marker (5′Δ5N-Pac) (Figure 2C) [54], suggesting that Pax2 (or Pax1) co-immunoprecipitated with anti-HA requires the HA-tagged ERK1 protein (Figure 11A). We further performed co-immunoprecipitation experiments with the encysting pPERK1m cultures. The anti-HA antibody did not immunoprecipitate ERK1m and Pax2 (or Pax1) in the pPERK1m cell line (Figure 11A). The results suggest an interaction between ERK1 and Pax2 (or Pax1) and the kinase domain is important for the interaction.

We also found that overexpression of ERK1 resulted in increased levels of the Pax2 and Pax1 proteins (Figure 11B). We also found a significant increase of the *pax2* and *pax1* mRNA levels in the ERK1-overexpressing cell line relative to the vector control cell line (Figure 11C). In addition, deletion of the predicted kinase domain resulted in decreased levels of the Pax2 and Pax1 proteins (Figure 11B). We also found a significant decrease of the *pax2* and *pax1* mRNA levels in the ERK1m-overexpressing cell line relative to the wild-type ERK1 overexpressing cell line (Figure 11C). As a control, similar levels of intensity of the giardial RAN protein (∼27 kDa) were detected by anti-RAN antibody (Figure 11B), and similar mRNA levels of the *ran* and *18S ribosomal RNA* genes were detected (Figure 11C). The results suggest that Pax2 or Pax1 may be a downstream component of a MAPK/ERK1 signaling pathway.

## Discussion

Despite the importance of cyst wall biogenesis during *Giardia* encystation, the underlying mechanism of gene regulation remains poorly understood. Pax family transcription factors have important roles in promoting organ development and cell differentiation in higher eukaryotes [46], [47], [48]. In the previous studies, we have identified a *pax1* gene whose expression increased during *Giardia* encystation [34]. Pax1 can bind AT-rich Inr elements in the promoter regions of key encystation-induced *cwp1-3* and *myb2* genes, and overexpression of Pax1 can induce the *cwp1-3* and *myb2* promoter activity, suggesting that Pax1 may be involved in co-ordinating their differential expression [34]. To gain insight into the function of Pax family transcription factors during *Giardia* encystation, we further investigated the role of giardial Pax2 in regulating *cwp* gene expression. Our results show that Pax2 localizes mainly to the nuclei (Figure 2E). We also found that overexpression of Pax2 increased the levels of the *cwp1-3* and *myb2* mRNA (Figure 8B and C). The levels of the CWP1 protein and cyst formation also increased in the Pax2 overexpressing cell line (Figure 8A and D). ChIP assays confirmed the association of Pax2 with its own promoter and the *cwp1-3* and *myb2* promoters (Figure 9C). In addition, deletion of C terminal paired domain or mutation of the basic residues of the paired domains resulted in a decrease of nuclear localization, DNA binding activity, and transactivation function of Pax2 on the expression of the *cwp1-3* and *myb2* genes (Figure 3, Figure 8, and Figure S1). Interestingly, the profiles of gene expression in the Pax2 and Pax1 overexpressing cells significantly overlap in the same direction (Figure 9B and Table S4). The results suggest that Pax2, like Pax1, may play an important role in induction of encystation. Duplication of functional redundant genes is frequently observed in eukaryotes [65]. Functional redundancy of Pax1 and Pax2 may have biological advantages, as increasing the supply of important transcription factors may help induction of encystation. In addition, if one gene is deleted or mutated, the other gene product can compensate its function, resulting in little phenotypic change.

The giardial promoters defined to date, including the *cwp* promoters, are short and contain AT-rich Inr elements [12], [13], [14], [25], [26], [27], [29], [30]. Deletion and mutation analysis of several gene promoters has provided the evidence that the AT-rich Inr elements are positive *cis*-acting elements and that they are important for basal promoter activity and transcription start site selection [26], [27], [30], [31]. Mutation of the AT-rich Inr element in the *cwp1* promoter might impair the binding of the Inr binding proteins to the Inr, leading to a downstream shift in transcription start site selection during vegetative growth [31]. During encystation, transcription start site selection of the *cwp1* promoter was shifted upstream, irrespective of mutations in the AT-rich Inr element. Therefore, the binding of some encystation-specific transcription factors to proximal upstream regions might be important for the selection of the upstream transcription start sites. Previously, we have identified several families of transcription factors in *G. lamblia* that may be involved in encystation, including Myb2, GARP-like protein 1, ARID1, WRKY, Pax1, and E2F1 [18], [29], [31], [32], [33], [34], [35]. We found that ARID1 and Pax1 can bind to the AT-rich Inr elements of the *cwp* promoters [31], [34]. In this study, we found that Pax2 can also bind to the AT-rich Inr elements of the *cwp* promoters (Figure 5 and Figure 6). Myb2, GARP-like protein 1, WRKY, and E2F1 can bind to the proximal upstream regions of the *cwp* promoters and their binding sequences are positive *cis*-acting elements [18], [29], [32], [35]. There may be an interaction of the transcription factors binding to the proximal upstream regions and the AT-rich Inr elements. This interaction may be required for promoter activity and accurate transcription start site selection.

Although the giardial Pax2 and Pax1 can be recognized as Pax proteins, they are divergent in sequence (Figure 1). The paired domain of giardial Pax2 (or Pax1) has 13.18% (or 19.85%) sequence identity and 29.46% (or 40.46%) similarity to that of human Pax6 [34], [58], suggesting that the giardial Pax1 is more like a Pax family protein than the giardial Pax2. The paired domain (or N-terminal region) of the giardial Pax2 only has 11.45% (or 14.53%) sequence identity and 28.24% (or 27.78%) sequence similarity to that of Pax1, suggesting that Pax2 have limited similarity with Pax1. The paired domains of both Pax2 and Pax1 have few of the conserved key contact residues and they have a predicted helix-turn-helix (HTH) structure similar to that of the human Pax family members (Figure 1 and data not shown). Mammalian Pax6 may bind to the G1 element which contains AT-rich sequence to activate the glucagon gene expression [66]. Our results indicate that the AT-rich Inr sequence may be important for binding of both Pax2 and Pax1 (Figure 5 and Figure 6) [34]. Further studies also indicate that both Pax2 and Pax1 can bind to the poly(A) sequence with a T, TT, TTT, or TC insertion, but not to poly(G) sequence (Figure 7A) [34]. We also found that Pax2 may bind strongly to a poly(A) sequence with a TC insertion (Figure 7A), and the binding activity is stronger than Pax1 [34]. The results suggest that both Pax1 and Pax2 may recognize variable AT-rich Inr sequences in different gene promoters and they may recognize different target sequences. We also found that the profiles of gene expression in the Pax2 and Pax1 overexpressing cells significantly overlap in the same direction. Oligonulceotide microarray assays confirmed the up-regulation of the *cwp1*, *cwp2*, *cwp3*, and *myb2* gene expression in the Pax2- or Pax1-overexpressing cell line to ∼1.55- to ∼7.99-fold (Figure 9A) [34]. We also found Pax2 or Pax1 may regulate epidermal growth factor-like cyst protein 3 gene (Table S4) [67]. Of the 185 (38) genes up-regulated in the Pax2 (Pax1) overexpressing cells, 19 genes were up-regulated in both the Pax2 and Pax1 overexpressing cells (Figure 9B and Table S4). Of the 172 (54) genes down-regulated in the Pax2 (Pax1) overexpressing cells, 30 genes were down-regulated in both the Pax2 and Pax1 overexpressing cells (Figure 9B and Table S4). About 50% and 56% of the genes that are up- or down-regulated in the Pax1 overexpressing cells overlap with those up or down regulated in the Pax2 overexpressing cells. About 10% and 17% of the genes that are up- or down-regulated in the Pax2 overexpressing cells overlap with those up or down regulated in the Pax1 overexpressing cells. Interestingly, of the 19 genes up-regulated in both the Pax2 and Pax1 overexpressing cells, 5 genes were also up-regulated during encystation (Table S4). Of the 30 genes down-regulated in both the Pax2 and Pax1 overexpressing cells, only 1 gene was also down-regulated during encystation and 2 genes were up-regulated during encystation (Table S4).

Many Pax proteins, including Pax1, 2, 3, 6 and 8, can recognize GTTCC sequence or a similar sequence (G/T)T(T/C)(C/A)(C/T)(G/C)(G/C) [42], [68]. Different Pax proteins have similar binding sequences, suggesting that different Pax proteins can recognize the same target genes. In addition, Pax proteins may have a high DNA-binding flexibility and they may bind to other sequences unrelated to GTTCC [69], [70]. Pax proteins may recognize different target sequences and regulate many different target genes through variable combinations of PAI and RED subdomains or homeodomain [69]. The variable sequence recognition ability may help Pax proteins to interact with other transcription factors [69]. We found that giardial Pax2 and Pax1 can bind to AT-rich sequence with a high flexibility (Figure 5, Figure 6, and Figure 7) [34], suggesting that both Pax2 and Pax1 may recognize AT-rich Inr elements of many different gene promoters and that it may interact with different transcription factors.

Pax2 and Pax1 can bind AT-rich Inr elements of both the constitutive *ran* gene and the encystation-induced *cwp1-3* and *myb2* genes, suggesting that Pax2 and Pax1 may be involved in transcriptional regulation of many different genes (Figure 6) [34]. Pax2 and Pax1 can not bind to the *18S ribosomal RNA* gene promoter which does not contain the AT-rich Inr element (Figure 6) [34]. It has been shown that the AT-rich Inr in the *ran* promoter is an important positive *cis*-acting element [26]. However, overexpressed Pax2 and Pax1 did not transactivate the *ran* promoter (Figure 8) [34]. The presence of the Pax2 and Pax1 binding sites/Inr in many gene promoters suggests that Pax2 and Pax1 may be involved in transcriptional regulation of many different genes. The great diversity of the promoter sequences among different genes suggests the flexibility of the requirements for transcription complex assembly in *G. lamblia*. Although Pax2 or Pax1 can also function as a transactivator, it may still need to cooperate with some other transcription factors that are induced during encystation to transactivate these cyst wall protein genes. In late-branching eukaryotes, Pax proteins regulate specific target genes by interacting with other classes of DNA binding proteins that occupy directly adjacent binding sites within the target promoter region. For, example, Pax5 can cooperate with Ets, a transcription factor containing a helix-turn-helix DNA-binding domain, to activate the *mb-1* gene in pre-B cell [71]. The paired domain of Pax3 interacts with the HMG domain of SOX10 to activate Mitf and Ret promoters [72]. Pax-6 could interact with c-Maf, a bZIP transcription factor, to activate the glucagon gene expression [66]. Therefore, it is possible that giardial Pax2 or Pax1 functions as an activator via association with some encystation-specific cofactors on the promoter context of encystation-induced genes.

Many important transcription factors involved in developmental regulation and in stress response have an autoregulation mechanism [73], [74]. Myb2 or WRKY has been found to be positively or negatively autoregulated to maintain its own protein levels and this is related to the presence of its binding sites in its own promoter region [18], [29], [33]. It has been shown that mammalian Pax proteins may be positively or negatively autoregulated by activating or inhibiting the activity of its own promoter [75]. We found that deletion of C terminal paired domain or mutation of the basic amino acids of the Pax2 paired domain resulted in a decrease of nuclear localization (Figure 3). The protein and mRNA levels of these Pax2 mutants increased significantly compared with that of wild type Pax2 (Figure 8), suggesting a negative autoregulation of the *pax2* gene. ChIP assays confirmed the association of Pax2 with its own promoter (Figure 9C). Similarly, Pax1 has also been found to be negatively autoregulated [34].

Two stretches of basic amino acids are present inside of the Pax2 paired domain (Figure 1), although they were not predicted as nuclear localization signals by PSORT program (http://psort.nibb.ac.jp/) [61]. We found that mutation of the N- or C-terminal one (residues 185–205 and 226–248, Pax2m1 and Pax2m2), resulted in a significant decrease of nuclear localization (Figure 3), suggesting that these two basic regions of the paired domain may play some role in the exclusively nuclear localization. Similarly, nuclear localization signal has been identified inside of the paired domains of human Pax6 [76]. In addition, we found a significant decrease of DNA binding activity and transactivation ability of Pax2m1 and Pax2m2 on the expression of the *cwp1-3* and *myb2* genes (Figure 8 and Figure S1). Because Pax2m1 and Pax2m2 were expressed at higher levels, their lower activity may be due to its lower ability to enter nuclei or to bind DNA. Because Pax2m1 was expressed more than Pax2m2, it may have higher DNA binding activity and transactivation ability. We also found that deletion of the paired domain and C-terminal 3 amino acids (residues 172–302, Pax2m3) resulted in a partial loss of nuclear localization, a decrease of DNA binding activity and transactivation ability on the expression of the *cwp1-3* and *myb2* genes (Figure 3, Figure 8, and Figure S1). Because Pax2m3 was expressed at higher levels, its lower activity may be due to its lower ability to enter nuclei or its lower ability to bind DNA. Our results suggest that the paired domain may be important for nuclear localization, DNA binding, and *in vivo* function. It is also possible that these specific regions of the paired domain may be positive regulatory regions for activation of transcription. Similarly, Pax1 has two basic regions in similar positions of the paired domain [34]. Similar results were also found in the Pax1 mutants, except that mutation of the N-terminal basic region did not affect nuclear localization, but still resulted in a decrease of DNA binding activity and transactivation ability [34].

Pax6 is highly expressed during development of the eyes and the central nervous system of zebrafish [77]. It can be phosphorylated by ERK and p38 kinase and phosphorylation can increase its transactivation activity, suggesting that Pax6 may act downstream of MAPK pathways [77]. One giardial MAPK, ERK1, exhibits a significantly increased kinase activity during early encystation and localizes to the giardial basal bodies [49], [78]. We found that the expression of the giardial ERK1 increased significantly during encystation and overexpression of ERK1 resulted in an increase of the *wrky*, *cwp1*, *cwp2*, and *myb2* mRNA levels, suggesting that the MAPK signaling cascades may modulate the amount and capability of WRKY for transactivation and/or DNA binding during encystation [18]. In this study, we found that ERK1 may function to phosphorylate Pax2 and Pax1 (Figure 10). We also found that overexpression of ERK1 resulted in an increase of the Pax2 and Pax2 mRNA and protein levels and that deletion of the predicted kinase domain of ERK1 resulted in a decreased of the Pax2 and Pax1 mRNA and protein levels (Figure 11), suggesting that Pax2 and Pax1 may be a downstream component of a MAPK/ERK1 signaling pathway. It is still possible that Pax proteins are not direct targets of the MAPK/ERK1. Further studies are awaited to elucidate which molecules in the MAPK/ERK1 signaling pathway are involved in phosphorylating the giardial Pax proteins.

Our study provides evidence for the involvement of Pax2 in DNA binding, transactivation of the *cwp1-3* and *myb2* genes, and induction of cyst formation of *G. lamblia*. Nine Pax family proteins play an important role in tissue and organ development in human [39]. Five Pax family proteins have been identified in *Caenorhabditis elegans* [79]. The presence of multiple Pax proteins in different organisms supports an important role of the Pax protein family. *Giardia* is a protozoan with a compact genome and relatively simplified machineries for DNA synthesis, transcription, and RNA processing. The presence of only two Pax proteins in *Giardia* also supports this view. The functional redundancy of the giardial Pax1 and Pax2 may have biological advantages, such as increasing the supply of specific transcription factors in induction of encystation. This is the first example of the presence of two functional analogues of transcription factors in *Giardia*. Our results may provide new insights into the progression of the control of gene expression from primitive to more complex eukaryotic cells and the evolution of eukaryotic DNA binding domain.

## Supporting Information

Figure S1 **Analysis of the DNA-binding domain of Pax2.** (A) Western blot analysis of recombinant Pax2 and Pax2m1-3 proteins. The Pax2 or Pax2m1-3 protein with a V5 tag at its C terminus was purified by affinity chromatography and then detected by anti-V5-HRP antibody in Western blots. (B-E) Reduction of DNA-binding ability of Pax2m1-3. Electrophoretic mobility shift assays were performed using purified Pax2 and Pax2m1-3, and specific probes, including cwp1-45/−1, cwp2-30/+8, cwp3-30/+10, and, ran-51/−20. The arrowhead indicates the shifted complex.
(PDF)

Table S1 **Oligonucleotides used in this study.**
(PDF)

Table S2 **Genes up or down regulated by Pax2 overexpression in microarray assays.**
(PDF)

Table S3 **Genes up or down regulated by Pax1 overexpression in microarray assays.**
(PDF)

Table S4 **Genes up or down regulated by both Pax1 and Pax2 overexpression in microarray assays.**
(PDF)
