# Supplementary figures and images for: Functional Redundancy of Two Pax-Like Proteins in Transcriptional Activation of Cyst Wall Protein Genes in Giardia lamblia
Source: PLoS One. 2012 Feb 15;7(2):e30614. doi: 10.1371/journal.pone.0030614 (PMC3280250; doi:10.1371/journal.pone.0030614)

Supplement Figure S1: Analysis of the DNA-binding domain of Pax2.

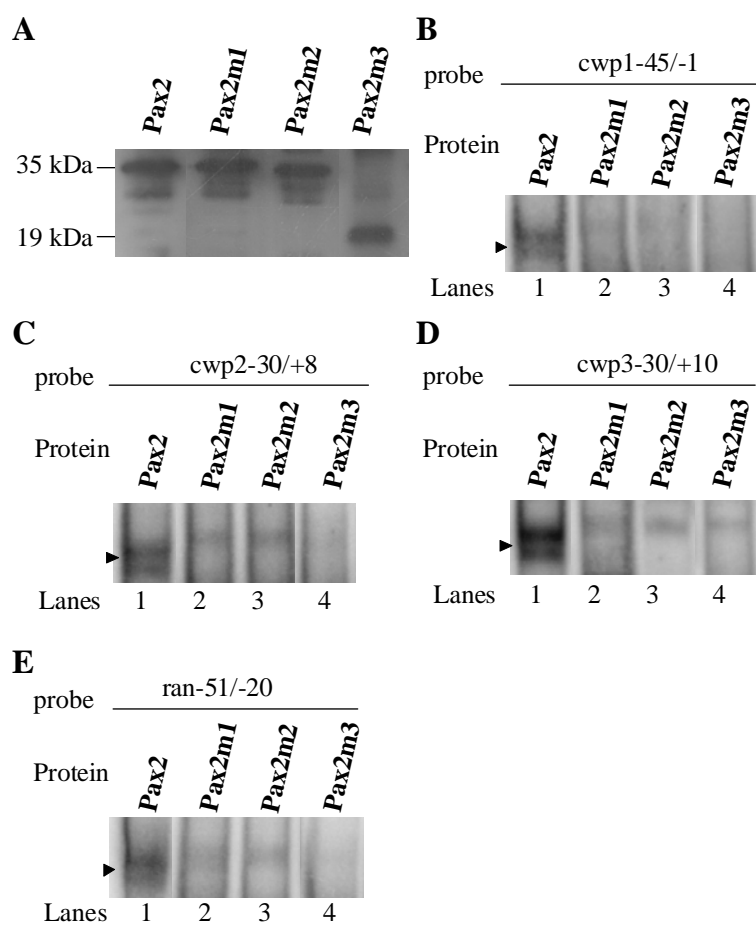

Supplement: Figure S1 — Analysis of the DNA-binding domain of Pax2. (A) Western blot analysis of recombinant Pax2 and Pax2m1-3 proteins. The Pax2 or Pax2m1-3 protein with a V5 tag at its C terminus was purified by affinity chromatography and then detected by anti-V5-HRP antibody in Western blots. (B-E) Reduction of DNA-binding ability of Pax2m1-3. Electrophoretic mobility shift assays were performed using purified Pax2 and Pax2m1-3, and specific probes, including cwp1-45/−1, cwp2-30/+8, cwp3-30/+10, and, ran-51/−20. The arrowhead indicates the shifted complex. (PDF) [file pone.0030614.s001.pdf]
