# Supplementary material for: Functional Redundancy of Two Pax-Like Proteins in Transcriptional Activation of Cyst Wall Protein Genes in Giardia lamblia
Source: PLoS One. 2012 Feb 15;7(2):e30614. doi: 10.1371/journal.pone.0030614 (PMC3280250; doi:10.1371/journal.pone.0030614)
Supplement: Table S1 — Oligonucleotides used in this study. (PDF) [file pone.0030614.s002.pdf]

Supplement Table S1. Oligonucleotides used in this study.

Name	Sequence (5’ to 3’)
Pax2F	CACCATGCAGGGGTCCTTCTATGGA
Pax2R	CTCATCGACCTGAGAGGACCT
Pax2HAR	AGCGTAATCTGGAACATCGTATGGGTA
cwp1F	ATGATGCTCGCTCTCCTT
cwp1R	TCAAGGCGGGGTGAGGCA
cwp2F	ATGATCGCAGCCCTTGTTCTA
cwp2R	CCTTCTGCGGACAATAGGCTT
cwp3F	ATGTTTTCTCTGCTTCTTCT
cwp3R	TCTGTAGTAGGGCGGCTGTA
myb2F	ATGTTACCGGTACCTTCTCAGC
myb2R	GGGTAGCTTCTCACGGGGAAG
ranF	ATGTCTGACCCAATCAGC
ranR	TCAATCATCGTCGGGAAG
18SrealF	AAGACCGCCTCTGTCAATCAA
18SrealR	GTTTACGGCCGGGAATACG
Pax2realF	CGCTGAGACTGAACCCATCAC
Pax2realR	CGCCAATCAGAGGCAGAGA
Pax2HAF	CATTGACACGGATGCTCGAT
cwp1realF	AACGCTCTCACAGGCTCCAT
cwp1realR	AGGTGGAGCTCCTTGAGAAATTG
cwp2realF	TAGGCTGCTTCCCACTTTTGAG
cwp2realR	CGGGCCCGCAAGGT
cwp3realF	GCAAATTGGATGCCAAACAA
cwp3realR	GACTCCGATCCAGTCGCAGTA
myb2realF	TCCCTAATGACGCCAAACG
myb2realR	AGCACGCAGAGGCCAAGT
ranrealF	TCGTCCTCGTCGGAAACAA
ranrealR	AACTGTCTGGGTGCGGATCT
Pax2XF	GGCGTCTAGACTTGCCTAAATGTTTATCCTG
Pax2MR	GGCGACGCGTCTCATCGACCTGAGAGGACCT
Pax2m1R	CGTATCTTCggcGTGCATATCCATGATggccagCCAATCAGAGGCAGAGAGcagggcGTGTGTGTCAACcagGGTGATGGG, mutated nucleotides are in lower case
Pax2m1F	CCCATCACCctgGTTGACACACACgccctgCTCTCTGCCTCTGATTGGctggccATCATGGATATGCACgccGAAGATACG, mutated region is shown in lower case

Pax2m2R	GCTTAGACCggcTGGcagCCCCAGcagggcGTTCTCATCcagATTATTTGGggcGATGCCggcTAGcagGATTATcagGTAAACCTG, mutated nucleotides are in lower case
Pax2m2F	CAGGTTTACctgATAATCctgCTAgccGGCATCgccCCAAATAATctgGATGAGAACgccctgCTGGGGctgCCAgccGGTCTAAGC, mutated region is shown in lower case
Pax2m3MR	GGCGACGCGTAGCGGATTGTGGATAACCATA
Pax2m3R	AGCGGATTGTGGATAACCATA
RanF	CACCATGTCTGACCCAATCAG
RanR	ATCATCGTCGGGAAGAGG
18S5F	CCAAAAAAGTGTGGTGCAGG
18S5R	GCCGGGCGCGGGCGCCGCGG
pax25F	GGAATCCGGGTGTTGGATGA pax25R
pax25R	AATAATAAAGCGGTTGGGTT
cwp15F	CAACGGCTTACTAAATCATTCTCTTG
cwp15R	TTCTGTGTTTCTTGATCTGAGAGTTGT
cwp25F	GAGCATGGGTTCTGACAAATAGTG
cwp25R	CATCAGTCTACTGTTTCTTTTTAGTTCATATCT
cwp35F	TTCGGGAGGGTCTGGGGGAG
cwp35R	ATCAGTAGTAACTTATTTTT
myb25F	TGCACTGTAGCGTTTCCATTTG
myb25R	ACTTACCCGTAATGGCGTTGAC
ran5F	GCCGCTTCAATGACAGATGC
ran5R	GCTACTCTCGGTTCCTGGGT
