# Supplementary material for: Functional Redundancy of Two Pax-Like Proteins in Transcriptional Activation of Cyst Wall Protein Genes in Giardia lamblia
Source: PLoS One. 2012 Feb 15;7(2):e30614. doi: 10.1371/journal.pone.0030614 (PMC3280250; doi:10.1371/journal.pone.0030614)
Supplement: Table S2 — Genes up or down regulated by Pax2 overexpression in microarray assays. (PDF) [file pone.0030614.s003.pdf]

Supplement Table S2. Genes up or down regulated by Pax2 overexpression in microarray assays.

Number	Annotation	Orf number	Fold change (pPPax2/5'Δ5N-Pac)[a]
1	Protein 21.1	7616	2.00 ($p<0.05$) *
2	Hypothetical protein	16078	2.00 ($p<0.05$)
3	Hypothetical protein	6946	2.01 ($p<0.05$)
4	Hypothetical protein	17603	2.02 ($p<0.05$)
5	Hypothetical protein	3063	2.03 ($p<0.05$)
6	Hypothetical protein	11976	2.03 ($p<0.05$)
7	Hypothetical protein	10568	2.03 ($p<0.05$)
8	Gmyb11	6417	2.04 ($p<0.05$)
9	Dynein heavy chain	17243	2.04 ($p<0.05$)
10	Hypothetical protein	3202	2.04 ($p<0.05$)
11	TM efflux prot	14247	2.05 ($p<0.05$)
12	Hypothetical protein	16287	2.06 ($p<0.05$)
13	Histone H4	135003	2.06 ($p<0.05$)
14	VSP	41472	2.07 ($p<0.05$)
15	Hypothetical protein	10527	2.07 ($p<0.05$)
16	Hypothetical protein	15240	2.08 ($p<0.05$)
17	Alpha-2 giardin	7796	2.08 ($p<0.05$)
18	Ubiquitin-conjugating enzyme E2-17 kDa	3978	2.08 ($p<0.05$)
19	CTP synthase /UTP-ammonia lyase	4507	2.08 ($p<0.05$)
20	Protein 21.1	13437	2.09 ($p<0.05$)
21	Kinase, NEK	137706	2.09 ($p<0.05$)
22	Hypothetical protein	17603	2.11 ($p<0.05$)
23	Hypothetical protein	16493	2.11 ($p<0.05$)
24	Hypothetical protein	1875	2.11 ($p<0.05$)
25	Hypothetical protein	10808	2.12 ($p<0.05$)
26	Hypothetical protein	15077	2.13 ($p<0.05$)
27	Caltractin	104685	2.14 ($p<0.05$)
28	SALP-1	4410	2.14 ($p<0.05$)
29	Protein 21.1	24590	2.14 ($p<0.05$)
30	Transitional endoplasmic reticulum ATPase	8524	2.16 ($p<0.05$)

31	VSP	137617	2.17 ($p<0.05$)
32	Gamma giardin	17230	2.18 ($p<0.05$)
33	Hypothetical protein	4984	2.18 ($p<0.05$)
34	Hypothetical protein	16367	2.18 ($p<0.05$)
35	Dynein light chain	27308	2.19 ($p<0.05$)
36	Phosphatidylinositol-4-phosphate 5-kinase, putative	2622	2.19 ($p<0.05$)
37	Hypothetical protein	8160	2.19 ($p<0.05$)
38	VSP	113304	2.20 ($p<0.05$)
39	Beta-giardin	4812	2.20 ($p<0.05$)
40	Hypothetical protein	11342	2.20 ($p<0.05$)
41	Kinase, CMGC CDK	16802	2.21 ($p<0.05$)
42	Hypothetical protein	112937	2.22 ($p<0.05$)
43	Hypothetical protein	135725	2.22 ($p<0.05$)
44	Giardia trophozoite antigen GTA-1	17090	2.22 ($p<0.05$)
45	Protein 21.1	16534	2.23 ($p<0.05$)
46	Hypothetical protein	10510	2.25 ($p<0.05$)
47	Protein 21.1	17585	2.25 ($p<0.05$)
48	Peptidyl-prolyl cis-trans isomerase B precursor	17000	2.25 ($p<0.05$)
49	Hypothetical protein	37258	2.26 ($p<0.05$)
50	Hypothetical protein	14471	2.26 ($p<0.05$)
51	Kinesin-14	13797	2.26 ($p<0.05$)
52	Hypothetical protein	16640	2.27 ($p<0.05$)
53	Carbamate Kinase	16453	2.27 ($p<0.05$)
54	G2/mitotic-specific cyclin B	3977	2.27 ($p<0.05$)
55	Hypothetical protein	31535	2.28 ($p<0.05$)
56	Endonuclease III	3595	2.28 ($p<0.05$)
57	VSP	114122	2.29 ($p<0.05$)
58	VSP, putative	96055	2.29 ($p<0.05$)
59	Hypothetical protein	10762	2.30 ($p<0.05$)
60	Phosphoacetylglucosamine mutase	16069	2.32 ($p<0.05$)
61	Translationally controlled tumor	6242	2.34 ($p<0.05$)

	protein-like protein		
62	Hypothetical protein	23630	2.35 ($p<0.05$)
63	Hypothetical protein	6330	2.36 ($p<0.05$)
64	High cysteine protein	94003	2.37 ($p<0.05$)
65	Phospholipid-transporting ATPase IA, putative	8182	2.38 ($p<0.05$)
66	Hypothetical protein	115669	2.38 ($p<0.05$)
67	Hypothetical protein	10238	2.41 ($p<0.05$)
68	Hypothetical protein	9068	2.42 ($p<0.05$)
69	Spindle pole protein, putative	13372	2.43 ($p<0.05$)
70	Hypothetical protein	87772	2.45 ($p<0.05$)
71	Hypothetical protein	16502	2.46 ($p<0.05$)
72	Protein 21.1	5188	2.46 ($p<0.05$)
73	Hypothetical protein	116865	2.48 ($p<0.05$)
74	Hypothetical protein	5800	2.49 ($p<0.05$)
75	Cyclin fold protein 1, putative	93721	2.51 ($p<0.05$)
76	Aldose reductase	7260	2.53 ($p<0.05$)
77	FKBP-type peptidyl-prolyl cis-trans isomerase	10570	2.55 ($p<0.05$)
78	Hypothetical protein	7598	2.55 ($p<0.05$)
79	Myb 1-like protein	8722	2.55 ($p<0.05$)
80	Hypothetical protein	17531	2.55 ($p<0.05$)
81	Protein 21.1	4846	2.57 ($p<0.05$)
82	Variant-specific surface protein	11690	2.59 ($p<0.05$)
83	Hypothetical protein	38432	2.59 ($p<0.05$)
84	VSP	112647	2.60 ($p<0.05$)
85	Hypothetical protein	2860	2.60 ($p<0.05$)
86	Hypothetical protein	3679	2.61 ($p<0.05$)
87	Hypothetical protein	10861	2.64 ($p<0.05$)
88	Hypothetical protein	14647	2.65 ($p<0.05$)
89	Hypothetical protein	22543	2.66 ($p<0.05$)
90	Deoxyribonuclease, TatD family	95789	2.66 ($p<0.05$)
91	ENC6 protein	102961	2.68 ($p<0.05$)

92	Serine palmitoyltransferase 2	14374	2.68 ($p<0.05$)
93	Hypothetical protein	112008	2.68 ($p<0.05$)
94	Glucosamine-6-phosphate deaminase	8245	2.69 ($p<0.05$)
95	Hypothetical protein	18722	2.71 ($p<0.05$)
96	Mitotic spindle checkpoint protein MAD2	100955	2.71 ($p<0.05$)
97	Hypothetical protein	11896	2.72 ($p<0.05$)
98	Hypothetical protein	3255	2.81 ($p<0.05$)
99	Furin precursor putative serine protease	2897	2.84 ($p<0.05$)
100	GTL3 aka MD0260	104866	2.85 ($p<0.05$)
101	High cysteine protein	17380	2.87 ($p<0.05$)
102	Hypothetical protein	10425	2.89 ($p<0.05$)
103	Hypothetical protein	17237	2.90 ($p<0.05$)
104	Hypothetical protein	116394	2.91 ($p<0.05$)
105	Hypothetical protein	33672	2.92 ($p<0.05$)
106	Hypothetical protein	5206	2.97 ($p<0.05$)
107	Hypothetical protein	23308	3.03 ($p<0.05$)
108	Hypothetical protein	2605	3.05 ($p<0.05$)
109	Hypothetical protein	10552	3.06 ($p<0.05$)
110	VSP	13402	3.06 ($p<0.05$)
111	Methyltransferase like 2	9528	3.07 ($p<0.05$)
112	Hypothetical protein	123980	3.11 ($p<0.05$)
113	UDP-N-acetylglucosamine pyrophosphorylase	16217	3.11 ($p<0.05$)
114	GlcNAc-PI synthesis protein	113610	3.15 ($p<0.05$)
115	Hypothetical protein	17012	3.16 ($p<0.05$)
116	Hypothetical protein	8960	3.19 ($p<0.05$)
117	Hypothetical protein	11050	3.19 ($p<0.05$)
118	Hypothetical protein	135270	3.24 ($p<0.05$)
119	VSP	102540	3.25 ($p<0.05$)
120	Sugar transport family protein	9046	3.26 ($p<0.05$)
121	Hypothetical protein	105786	3.27 ($p<0.05$)

122	Hypothetical protein	91187	3.28 (p<0.05)
123	ABC transporter	42048	3.37 (p<0.05)
124	CEGP1 protein	17120	3.37 (p<0.05)
125	Hypothetical protein	117989	3.40 (p<0.05)
126	C4 group specific protein	13747	3.40 (p<0.05)
127	Hypothetical protein	11120	3.55 (p<0.05)
128	Retinoic acid induced 17-like protein	11930	3.59 (p<0.05)
129	UDP-glucose 4-epimerase	7982	3.59 (p<0.05)
130	Hypothetical protein	36883	3.64 (p<0.05)
131	Cyst wall protein 2	5435	3.64 (p<0.05)
132	VSP	112048	3.67 (p<0.05)
133	Hypothetical protein	32657	3.74 (p<0.05)
134	Hypothetical protein	7374	3.75 (p<0.05)
135	Flap structure-specific endonuclease	16953	3.77 (p<0.05)
136	VSP	124980	3.77 (p<0.05)
137	Ceramide glucosyltransferase	11642	3.78 (p<0.05)
138	Hypothetical protein	2692	3.78 (p<0.05)
139	VSP	8338	3.79 (p<0.05)
140	Hypothetical protein	125106	3.86 (p<0.05)
141	Hypothetical protein	29130	3.99 (p<0.05)
142	VSP	137612	4.10 (p<0.05)
143	Hypothetical protein	8505	4.15 (p<0.05)
144	Fatty acid elongase 1	92729	4.19 (p<0.05)
145	Adenylate cyclase	14367	4.24 (p<0.05)
146	Hypothetical protein	7353	4.26 (p<0.05)
147	Hypothetical protein	19870	4.38 (p<0.05)
148	High cysteine membrane protein Group 4	114930	4.54 (p<0.05)
149	VSP	111873	4.62 (p<0.05)
150	VSP, putative	92835	4.65 (p<0.05)
151	Hypothetical protein	27652	4.76 (p<0.05)
152	Hypothetical protein	14690	5.02 (p<0.05)

153	Zinc finger domain	2116	5.09 ($p<0.05$)
154	Hypothetical protein	3731	5.38 ($p<0.05$)
155	Hypothetical protein	28112	5.84 ($p<0.05$)
156	Variant-specific surface protein	9276	6.33 ($p<0.05$)
157	VSP	41539	6.38 ($p<0.05$)
158	High cysteine membrane protein Group 1	10659	6.40 ($p<0.05$)
159	Hypothetical protein	10763	6.53 ($p<0.05$)
160	VSP	137611	6.98 ($p<0.05$)
161	High cysteine membrane protein Group 1	11309	7.02 ($p<0.05$)
162	Hypothetical protein	23934	7.46 ($p<0.05$)
163	Cyst wall protein 1	5638	8.24 ($p<0.05$)
164	Glucose 6-phosphate N-acetyltransferase	14259	8.31 ($p<0.05$)
165	Hypothetical protein	16622	8.87 ($p<0.05$)
166	VSP	135882	9.69 ($p<0.05$)
167	VSP	32916	9.72 ($p<0.05$)
168	VSP	135919	9.86 ($p<0.05$)
169	VSP	40630	10.42 ($p<0.05$)
170	VSP	97233	10.46 ($p<0.05$)
171	VSP	115085	11.08 ($p<0.05$)
172	VSP	26590	11.84 ($p<0.05$)
173	VSP	13520	12.27 ($p<0.05$)
174	VSP	14307	13.81 ($p<0.05$)
175	Variant-specific surface protein VSP4A1 precursor	101498	14.28 ($p<0.05$)
176	VSP	118900	14.31 ($p<0.05$)
177	VSP, putative	103916	14.82 ($p<0.05$)
178	VSP	111874	16.23 ($p<0.05$)
179	VSP	137610	16.80 ($p<0.05$)
180	VSP	105983	17.05 ($p<0.05$)
181	VSP	15206	17.51 ($p<0.05$)
182	VSP	41476	19.40 ($p<0.05$)

183	VSP	137620	28.46 ($p<0.05$)
184	VSP	113093	32.67 ($p<0.05$)
185	High cysteine membrane protein Group 1	25816	64.30 ($p<0.05$)
186	VSP with INR	113439	0.07 ($p<0.05$)
187	VSP	13390	0.10 ($p<0.05$)
188	Hypothetical protein	4439	0.21 ($p<0.05$)
189	High cysteine membrane protein Group 4	114089	0.22 ($p<0.05$)
190	VSP with INR	113797	0.23 ($p<0.05$)
191	Hypothetical protein	39159	0.24 ($p<0.05$)
192	Hypothetical protein	9552	0.25 ($p<0.05$)
193	Hypothetical protein	26727	0.26 ($p<0.05$)
194	Hypothetical protein	28566	0.26 ($p<0.05$)
195	Hypothetical protein	93743	0.26 ($p<0.05$)
196	VSP	111903	0.29 ($p<0.05$)
197	VSP	99743	0.29 ($p<0.05$)
198	VSP with INR	40592	0.29 ($p<0.05$)
199	Hypothetical protein	20020	0.30 ($p<0.05$)
200	VSP, putative	118181	0.30 ($p<0.05$)
201	Hypothetical protein	36122	0.30 ($p<0.05$)
202	Hypothetical protein	102575	0.30 ($p<0.05$)
203	Hypothetical protein	112017	0.30 ($p<0.05$)
204	Hypothetical protein	114044	0.30 ($p<0.05$)
205	VSP, putative	118133	0.31 ($p<0.05$)
206	High cysteine membrane protein Group 3	114891	0.31 ($p<0.05$)
207	Hypothetical protein	14637	0.31 ($p<0.05$)
208	Hypothetical protein	6666	0.31 ($p<0.05$)
209	Hypothetical protein	14637	0.31 ($p<0.05$)
210	Hypothetical protein	99071	0.33 ($p<0.05$)
211	Pyruvate-flavodoxin oxidoreductase	114609	0.34 ($p<0.05$)
212	Hypothetical protein	114043	0.34 ($p<0.05$)
213	Hypothetical protein	5613	0.34 ($p<0.05$)

214	VSP	137607	0.34 (p<0.05)
215	Hypothetical protein	112018	0.34 (p<0.05)
216	Hypothetical protein	99726	0.34 (p<0.05)
217	High cysteine membrane protein TMK-like	113416	0.35 (p<0.05)
218	Hypothetical protein	17332	0.35 (p<0.05)
219	DNA-damage inducible protein DDI1-like	7718	0.36 (p<0.05)
220	VSP	113954	0.36 (p<0.05)
221	Kinase, NEK	7103	0.36 (p<0.05)
222	Axoneme-associated protein GASP-180	13475	0.36 (p<0.05)
223	Cathepsin L precursor	9548	0.37 (p<0.05)
224	VSP	137606	0.37 (p<0.05)
225	Pyruvate-flavodoxin oxidoreductase	17063	0.38 (p<0.05)
226	Hypothetical protein	18559	0.38 (p<0.05)
227	Hypothetical protein	92919	0.38 (p<0.05)
228	Hypothetical protein	16293	0.39 (p<0.05)
229	Hypothetical protein	111906	0.39 (p<0.05)
230	VSP	116477	0.39 (p<0.05)
231	High cysteine membrane protein Group 3	112126	0.39 (p<0.05)
232	Hypothetical protein	23767	0.39 (p<0.05)
233	Ubiquitin-conjugating enzyme E2-17 kDa	12950	0.40 (p<0.05)
234	Serine/Threonine-protei n kinase pkwA, putative	9173	0.40 (p<0.05)
235	Hypothetical protein	113673	0.40 (p<0.05)
236	Hypothetical protein	135970	0.40 (p<0.05)
237	High cysteine protein	87706	0.40 (p<0.05)
238	Hypothetical protein	88888	0.40 (p<0.05)
239	Kinase, NEK	42657	0.40 (p<0.05)
240	IFT complex B	40995	0.40 (p<0.05)
241	VSP	41349	0.40 (p<0.05)
242	Dynein heavy chain	111950	0.40 (p<0.05)

243	NADP-specific glutamate dehydrogenase	21942	0.40 (p<0.05)
244	Hypothetical protein	116090	0.41 (p<0.05)
245	Cysteinyl-tRNA synthetase	5867	0.41 (p<0.05)
246	VSP	98310	0.41 (p<0.05)
247	Hypothetical protein	17266	0.41 (p<0.05)
248	Glyceraldehyde 3-phosphate dehydrogenase	17043	0.41 (p<0.05)
249	VSP	115796	0.41 (p<0.05)
250	Hypothetical protein	120486	0.41 (p<0.05)
251	Hypothetical protein	2366	0.41 (p<0.05)
252	Hypothetical protein	35487	0.41 (p<0.05)
253	Hypothetical protein	111809	0.42 (p<0.05)
254	VSP	98058	0.42 (p<0.05)
255	Dynein heavy chain	101138	0.42 (p<0.05)
256	Hypothetical protein	2355	0.42 (p<0.05)
257	Hypothetical protein	16232	0.42 (p<0.05)
258	Axoneme-associated protein GASP-180	137716	0.42 (p<0.05)
259	Hypothetical protein	104062	0.42 (p<0.05)
260	Protein 21.1	17562	0.42 (p<0.05)
261	Kinase, NEK-frag	16826	0.43 (p<0.05)
262	VSP	101410	0.43 (p<0.05)
263	Hypothetical protein	115613	0.43 (p<0.05)
264	Hypothetical protein	28464	0.43 (p<0.05)
265	VSP	117204	0.43 (p<0.05)
266	Hypothetical protein	16751	0.43 (p<0.05)
267	Glycerol Kinase	8173	0.43 (p<0.05)
268	Hypothetical protein	9551	0.43 (p<0.05)
269	Hypothetical protein	127181	0.43 (p<0.05)
270	High cysteine membrane protein Group 1	7715	0.43 (p<0.05)
271	Hypothetical protein	23560	0.44 (p<0.05)
272	VSP S8	137604	0.44 (p<0.05)

273	Hypothetical protein	8055	0.44 (p<0.05)
274	Protein 21.1	14834	0.44 (p<0.05)
275	High cysteine membrane protein Group 1	15317	0.44 (p<0.05)
276	Kinase, NEK	16122	0.44 (p<0.05)
277	Phosphoenolpyruvate carboxyKinase	10623	0.44 (p<0.05)
278	Kinase, NEK-frag	7183	0.45 (p<0.05)
279	Hypothetical protein	26369	0.45 (p<0.05)
280	Zinc finger protein	106320	0.45 (p<0.05)
281	Hypothetical protein	28994	0.45 (p<0.05)
282	Hypothetical protein	32543	0.45 (p<0.05)
283	tRNA delta2-isopentenylpyrop hosphate transferase	17480	0.45 (p<0.05)
284	VSP, putative	134711	0.46 (p<0.05)
285	GARP-like protein 4	33232	0.46 (p<0.05)
286	Hypothetical protein	87955	0.46 (p<0.05)
287	Kinase, NEK	93221	0.46 (p<0.05)
288	Hypothetical protein	2178	0.46 (p<0.05)
289	VSP with INR	119707	0.46 (p<0.05)
290	Hypothetical protein	22814	0.46 (p<0.05)
291	MutT/nudix family protein	4204	0.46 (p<0.05)
292	Hypothetical protein	16065	0.47 (p<0.05)
293	CXC-rich protein	14225	0.47 (p<0.05)
294	Hypothetical protein	33978	0.47 (p<0.05)
295	Nucleotide-binding protein 1	14604	0.47 (p<0.05)
296	Hypothetical protein	101278	0.47 (p<0.05)
297	Hypothetical protein	14615	0.47 (p<0.05)
298	Kinase, NEK	101307	0.47 (p<0.05)
299	Hypothetical protein	98760	0.47 (p<0.05)
300	Kinase, NEK	86934	0.47 (p<0.05)
301	VSP	41401	0.47 (p<0.05)
302	Hypothetical protein	27583	0.47 (p<0.05)
303	Metal-dependent	6497	0.47 (p<0.05)

	hydrolase		
304	Hypothetical protein	3603	0.47 (p<0.05)
305	Variant-specific surface protein	6101	0.47 (p<0.05)
306	Hypothetical protein	3916	0.47 (p<0.05)
307	Centromere/microtubule binding protein CBF5	16311	0.47 (p<0.05)
308	Hypothetical protein	2201	0.47 (p<0.05)
309	Hypothetical protein	13272	0.47 (p<0.05)
310	Phosphomannomutase-2	11448	0.47 (p<0.05)
311	Hypothetical protein	11866	0.47 (p<0.05)
312	Hypothetical protein	17102	0.47 (p<0.05)
313	Hypothetical protein	39766	0.47 (p<0.05)
314	Dynein heavy chain	94440	0.47 (p<0.05)
315	Hypothetical protein	39607	0.48 (p<0.05)
316	Hypothetical protein	106941	0.48 (p<0.05)
317	Tenascin precursor	114815	0.48 (p<0.05)
318	Hypothetical protein	16414	0.48 (p<0.05)
319	Alcohol dehydrogenase lateral transfer candidate	3593	0.48 (p<0.05)
320	Hypothetical protein	22008	0.48 (p<0.05)
321	VSP	134710	0.48 (p<0.05)
322	High cysteine protein	14783	0.48 (p<0.05)
323	High cysteine protein	102180	0.48 (p<0.05)
324	Hypothetical protein	33855	0.48 (p<0.05)
325	Bardet-Biedl syndrome 4 protein-like protein	10529	0.48 (p<0.05)
326	High cysteine protein	112604	0.48 (p<0.05)
327	VSP	113024	0.48 (p<0.05)
328	Hypothetical protein	29774	0.48 (p<0.05)
329	VSP	113211	0.48 (p<0.05)
330	Microsomal signal peptidase 18 kDa subunit	9174	0.48 (p<0.05)
331	VSP	34357	0.48 (p<0.05)
332	Ribosomal protein L15	8001	0.48 (p<0.05)
333	Hypothetical protein	123336	0.49 (p<0.05)

334	VSP AS8	13194	0.49 (*p*<0.05)
335	Hypothetical protein	32578	0.49 (*p*<0.05)
336	Hypothetical protein	3855	0.49 (*p*<0.05)
337	Acidic ribosomal protein P0	17054	0.49 (*p*<0.05)
338	Tenascin-like	16833	0.49 (*p*<0.05)
339	VSP	40591	0.49 (*p*<0.05)
340	Protein 21.1	17285	0.49 (*p*<0.05)
341	Methyltransferase, putative	103058	0.49 (*p*<0.05)
342	VSP	114672	0.49 (*p*<0.05)
343	Hypothetical protein	105343	0.49 (*p*<0.05)
344	Hypothetical protein	9705	0.49 (*p*<0.05)
345	DINP protein human, muc B family	16810	0.49 (*p*<0.05)
346	GARP-like protein 3	9154	0.49 (*p*<0.05)
347	Hypothetical protein	96732	0.49 (*p*<0.05)
348	Hypothetical protein	123280	0.50 (*p*<0.05)
349	Kinase, NEK	12240	0.50 (*p*<0.05)
350	VSP	90215	0.50 (*p*<0.05)
351	Kinase, CDC7	112076	0.50 (*p*<0.05)
352	Hypothetical protein	13539	0.50 (*p*<0.05)
353	High cysteine membrane protein Group 1	91707	0.50 (*p*<0.05)
354	Ciliary dynein heavy chain 11	42285	0.50 (*p*<0.05)
355	Inorganic polyphosphate/ATP-NAD kinase, putative	17316	0.50 (*p*<0.05)
356	Methyltransferase, putative	100887	0.50 (*p*<0.05)
357	VSP	40571	0.50 (*p*<0.05)

[a]The 5’Δ5N-Pac and pPPax2 stable transfectants were cultured in growth medium for 24 h and then subjected to microarray assays.

*Fold changes in mRNA expression are shown as the ratio of transcript levels in the

pPPax2 cell line relative to the 5'Δ5N-Pac cell line. *p* values were determined for groups in which the average means changed by a factor of ≥2.0 or ≤0.5.
