# Supplementary material for: Functional Redundancy of Two Pax-Like Proteins in Transcriptional Activation of Cyst Wall Protein Genes in Giardia lamblia
Source: PLoS One. 2012 Feb 15;7(2):e30614. doi: 10.1371/journal.pone.0030614 (PMC3280250; doi:10.1371/journal.pone.0030614)
Supplement: Table S3 — Genes up or down regulated by Pax1 overexpression in microarray assays. (PDF) [file pone.0030614.s004.pdf]

Supplement Table S3. Genes up or down regulated by Pax1 overexpression in microarray assays.

Number	Annotation	Orf number	Fold change (pPPax1/5’Δ5N-Pac)[a]
1	Protein 21.1	15965	2.01 (*p*<0.05) *
2	Hypothetical protein	8960	2.01 (*p*<0.05)
3	Heat shock protein HSP 90-alpha	13864	2.03 (*p*<0.05)
4	VSP with INR	101074	2.04 (*p*<0.05) *
5	TM efflux prot	14247	2.07 (*p*<0.05)
6	Hypothetical protein	91187	2.08 (*p*<0.05)
7	Protein disulfide isomerase PDI4	103713	2.11 (*p*<0.05)
8	Hypothetical protein	10808	2.11 (*p*<0.05)
9	Deoxyribonuclease, TatD family	95789	2.12 (*p*<0.05)
10	VSP	112048	2.13 (*p*<0.05)
11	Hypothetical protein	14997	2.14 (*p*<0.05)
12	Hypothetical protein	10510	2.14 (*p*<0.05)
13	Protein 21.1	102813	2.18 (*p*<0.05)
14	VSP	8338	2.19 (*p*<0.05)
15	VSP	137610	2.20 (*p*<0.05)
16	Hypothetical protein	10552	2.21 (*p*<0.05)
17	Hypothetical protein	22502	2.22 (*p*<0.05)
18	Hypothetical protein	38432	2.35 (*p*<0.05)
19	VSP	15206	2.36 (*p*<0.05)
20	Hypothetical protein	15125	2.39 (*p*<0.05)
21	Heat-shock protein, putative	16412	2.39 (*p*<0.05)
22	Protein disulfide isomerase PDI3	14670	2.44 (*p*<0.05)
23	Hypothetical protein	20315	2.44 (*p*<0.05)
24	Protein 21.1	4846	2.45 (*p*<0.05)
25	VSP	41476	2.51 (*p*<0.05)
26	Hypothetical protein	15532	2.58 (*p*<0.05)
27	Heat shock protein HSP 90-alpha	98054	2.64 (*p*<0.05)
28	Hypothetical protein	16424	2.76 (*p*<0.05)

29	Hypothetical protein	116865	2.85 ($p<0.05$)
30	Cyst wall protein 1	5638	2.99 ($p<0.05$)
31	VSP	137617	3.03 ($p<0.05$)
32	High cysteine protein	17380	3.29 ($p<0.05$)
33	Hypothetical protein	9605	3.36 ($p<0.05$)
34	C4 group specific protein	13747	3.40 ($p<0.05$)
35	Hypothetical protein	10294	3.50 ($p<0.05$)
36	Hypothetical protein	112811	3.82 ($p<0.05$)
37	VSP	137612	4.41 ($p<0.05$)
38	VSP	137620	5.06 ($p<0.05$)
39	VSP	13390	0.11 ($p<0.05$)
40	VSP with INR	113439	0.17 ($p<0.05$)
41	VSP	101765	0.24 ($p<0.05$)
42	VSP with INR	119707	0.30 ($p<0.05$)
43	VSP	137606	0.30 ($p<0.05$)
44	Hypothetical protein	99726	0.32 ($p<0.05$)
45	VSP	40571	0.32 ($p<0.05$)
46	VSP	90215	0.32 ($p<0.05$)
47	High cysteine membrane protein Group 1	7715	0.33 ($p<0.05$)
48	VSP	98058	0.34 ($p<0.05$)
49	VSP	34357	0.34 ($p<0.05$)
50	Hypothetical protein	28566	0.36 ($p<0.05$)
51	High cysteine membrane protein Group 1	15317	0.36 ($p<0.05$)
52	Dynein heavy chain	101138	0.36 ($p<0.05$)
53	VSP with INR	113450	0.38 ($p<0.05$)
54	VSP	115796	0.39 ($p<0.05$)
55	Pyruvate-flavodoxin oxidoreductase	114609	0.39 ($p<0.05$)
56	Hypothetical protein	36122	0.40 ($p<0.05$)
57	VSP	137723	0.40 ($p<0.05$)
58	VSP	32933	0.40 ($p<0.05$)
59	Pyruvate-flavodoxin oxidoreductase	17063	0.41 ($p<0.05$)

60	High cysteine protein	6372	0.41 (p<0.05)
61	Hypothetical protein	17332	0.42 (p<0.05)
62	VSP, putative	118181	0.42 (p<0.05)
63	VSP AS8	13194	0.42 (p<0.05)
64	VSP	40591	0.42 (p<0.05)
65	Tenascin precursor	114815	0.42 (p<0.05)
66	VSP	114672	0.43 (p<0.05)
67	Hypothetical protein	101278	0.43 (p<0.05)
68	VSP	113357	0.43 (p<0.05)
69	VSP	115047	0.44 (p<0.05)
70	Hypothetical protein	3731	0.44 (p<0.05)
71	VSP with INR	14586	0.44 (p<0.05)
72	Pyruvate, phosphate diKinase,	9909	0.45 (p<0.05)
73	VSP with INR	40592	0.46 (p<0.05)
74	Hypothetical protein	93278	0.46 (p<0.05)
75	Hypothetical protein	113038	0.46 (p<0.05)
76	Hypothetical protein	15573	0.46 (p<0.05)
77	Chorein	87358	0.47 (p<0.05)
78	Dynein heavy chain	17265	0.47 (p<0.05)
79	Coiled-coil protein	17249	0.47 (p<0.05)
80	VSP, putative	118133	0.47 (p<0.05)
81	VSP	111936	0.47 (p<0.05)
82	Acyl-CoA synthetase	86511	0.48 (p<0.05)
83	Dynein heavy chain	111950	0.48 (p<0.05)
84	Hypothetical protein	3269	0.48 (p<0.05)
85	Hypothetical protein	1742	0.48 (p<0.05)
86	VSP	113242	0.48 (p<0.05)
87	Hypothetical protein	123336	0.48 (p<0.05)
88	AAA family ATPase	16867	0.49 (p<0.05)
89	Glutaminyl-tRNA synthetase	86681	0.49 (p<0.05)
90	VSP	111933	0.49 (p<0.05)
91	Hypothetical protein	112017	0.49 (p<0.05)
92	Dynein heavy chain	94440	0.50 (p<0.05)

[a]The 5’Δ5N-Pac and pPPax1 stable transfectants were cultured in growth medium for 24 h and then subjected to microarray assays.

*Fold changes in mRNA expression are shown as the ratio of transcript levels in the pPPax1 cell line relative to the 5’Δ5N-Pac cell line. *p* values were determined for groups in which the average means changed by a factor of ≥2.0 or ≤0.5.
