# Supplementary material for: Functional Redundancy of Two Pax-Like Proteins in Transcriptional Activation of Cyst Wall Protein Genes in Giardia lamblia
Source: PLoS One. 2012 Feb 15;7(2):e30614. doi: 10.1371/journal.pone.0030614 (PMC3280250; doi:10.1371/journal.pone.0030614)
Supplement: Table S4 — Genes up or down regulated by both Pax1 and Pax2 overexpression in microarray assays. (PDF) [file pone.0030614.s005.pdf]

Supplement Table S4. Genes up or down regulated by both Pax1 and Pax2 overexpression in microarray assays.

Number	Annotation	Orf number	Fold change (pPPax1/5’Δ5N-Pac)[a]	Fold change (pPPax2/5’Δ5N-Pac)[b]	Fold change (Enc/Veg)[c]
1	VSP	137620	5.06 (p<0.05) *	27.29 (p<0.05)	0.70
2	VSP	41476	2.51 (p<0.05)	19.30 (p<0.05)	2.24 (p<0.05)
3	VSP	137610	2.20 (p<0.05)	16.57 (p<0.05)	0.72
4	Cyst wall protein 1	5638	2.99 (p<0.05)	7.99 (p<0.05)	47.88 (p<0.05)
5	VSP	137612	4.41 (p<0.05)	4.09 (p<0.05)	1.05
6	VSP	8338	2.19 (p<0.05)	3.63 (p<0.05)	1.75
7	VSP	112048	2.13 (p<0.05)	3.56 (p<0.05)	1.82
8	C4 group specific protein	13747	3.40 (p<0.05)	3.35 (p<0.05)	2.97 (p<0.05)
9	Hypothetical protein	8960	2.01 (p<0.05)	3.11 (p<0.05)	1.88
10	Hypothetical protein	91187	2.08 (p<0.05)	3.03 (p<0.05)	1.00
11	Hypothetical protein	10552	2.21 (p<0.05)	2.95 (p<0.05)	18.71 (p<0.05)
12	High cysteine protein	17380	3.29 (p<0.05)	2.83 (p<0.05)	0.62
13	Protein 21.1	4846	2.45 (p<0.05)	2.55 (p<0.05)	5.42 (p<0.05)
14	Hypothetical protein	38432	2.35 (p<0.05)	2.51 (p<0.05)	1.29
15	Hypothetical protein	116865	2.85 (p<0.05)	2.42 (p<0.05)	0.76
16	VSP	137617	3.03 (p<0.05)	2.13 (p<0.05)	0.93
17	Hypothetical protein	10510	2.14 (p<0.05)	2.10 (p<0.05)	1.80
18	Hypothetical protein	10808	2.11 (p<0.05)	2.10 (p<0.05)	0.60
19	TM efflux prot	14247	2.07 (p<0.05)	2.03 (p<0.05)	0.82
20	VSP with INR	113439	0.17 (p<0.05)	0.07 (p<0.05)	0.80
21	VSP	13390	0.11 (p<0.05)	0.10 (p<0.05)	1.00
22	VSP with INR	40592	0.46 (p<0.05)	0.30 (p<0.05)	0.95
23	VSP, putative	118181	0.42 (p<0.05)	0.31 (p<0.05)	1.08
24	Hypothetical protein	112017	0.50 (p<0.05)	0.31 (p<0.05)	0.46 (p<0.05)
25	Hypothetical protein	28566	0.36 (p<0.05)	0.32 (p<0.05)	1.00

26	VSP, putative	118133	0.48 (p<0.05)	0.32 (p<0.05)	1.09
27	Pyruvate-flavodoxin oxidoreductase	114609	0.40 (p<0.05)	0.34 (p<0.05)	1.83
28	Hypothetical protein	99726	0.32 (p<0.05)	0.35 (p<0.05)	0.93
29	Hypothetical protein	17332	0.42 (p<0.05)	0.36 (p<0.05)	0.82
30	VSP	137606	0.31 (p<0.05)	0.38 (p<0.05)	1.08
31	Pyruvate-flavodoxin oxidoreductase	17063	0.41 (p<0.05)	0.39 (p<0.05)	2.61 (p<0.05)
32	Dynein heavy chain	111950	0.48 (p<0.05)	0.42 (p<0.05)	1.10
33	Dynein heavy chain	101138	0.37 (p<0.05)	0.42 (p<0.05)	0.85
34	VSP	98058	0.34 (p<0.05)	0.45 (p<0.05)	0.96
35	High cysteine membrane protein Group 1	7715	0.33 (p<0.05)	0.45 (p<0.05)	1.29
36	High cysteine membrane protein Group 1	15317	0.37 (p<0.05)	0.45 (p<0.05)	2.13 (p<0.05)
37	VSP with INR	119707	0.30 (p<0.05)	0.47 (p<0.05)	1.03
38	VSP	115796	0.39 (p<0.05)	0.48 (p<0.05)	1.28
39	Hypothetical protein	101278	0.43 (p<0.05)	0.49 (p<0.05)	1.54
40	Hypothetical protein	123336	0.49 (p<0.05)	0.49 (p<0.05)	0.86
41	VSP AS8	13194	0.42 (p<0.05)	0.49 (p<0.05)	0.98
42	VSP	90215	0.33 (p<0.05)	0.49 (p<0.05)	0.98
43	Hypothetical protein	36122	0.40 (p<0.05)	0.50 (p<0.05)	1.00
44	Dynein heavy chain	94440	0.50 (p<0.05)	0.50 (p<0.05)	1.02
45	Tenascin precursor ([#]EGFCP3)	114815	0.43 (p<0.05)	0.50 (p<0.05)	0.99
46	VSP	34357	0.34 (p<0.05)	0.50 (p<0.05)	0.98
47	VSP	40591	0.43 (p<0.05)	0.50 (p<0.05)	1.04
48	VSP	114672	0.43 (p<0.05)	0.50 (p<0.05)	1.08
49	VSP	40571	0.33 (p<0.05)	0.50 (p<0.05)	1.00

[a]The 5’Δ5N-Pac and pPPax1 stable transfectants were cultured in growth medium for 24 h and

then subjected to microarray assays.

[b]The 5’Δ5N-Pac and pPPax2 stable transfectants were cultured in growth medium for 24 h and then subjected to microarray assays.

[c]The wild-type non-transfected WB cells were cultured in growth (Veg, vegetative growth) or encystation medium for 24 h (Enc, encystation) and then subjected to microarray assays.

*Fold changes in mRNA expression are shown as the ratio of transcript levels in the pPPax1 cell line relative to the 5’Δ5N-Pac cell line. *p* values were determined for groups in which the average means changed by a factor of ≥2.0 or ≤0.5.

[#]Epidermal Growth Factor-like Cyst Protein 3 [67].
